# Exploratory multi-omics analysis suggests context-dependent immune responses to vitamin D_3_ supplementation in PBMCs of multiple sclerosis patients

**DOI:** 10.3389/fimmu.2026.1845005

**Published:** 2026-07-31

**Authors:** Tanya Tripathi, Emilia Gospodarska, Rohat Geran, Pia Sophie Sperber, Tanja Schmitz-Hübsch, Friedemann Paul, Carsten Carlberg

**Affiliations:** 1 InLife Institute of Animal Reproduction and Food Research, Polish Academy of Sciences, Olsztyn, Poland; 2 Experimental and Clinical Research Center, a Cooperation between the Max-Delbrück-Center for Molecular Medicine in the Helmholtz Association and the Charité-Universitätsmedizin Berlin, Berlin, Germany; 3 Charité-Universitätsmedizin Berlin, Corporate Member of Freie Universität Berlin and Humboldt-Universität zu Berlin, Berlin, Germany; 4 Max-Delbrück-Center for Molecular Medicine in the Helmholtz Association, Berlin, Germany; 5 Department of Neurology with Experimental Neurology, Charité-Universitätsmedizin Berlin, Corporate Member of Freie Universität Berlin and Humboldt Universität zu Berlin, Berlin, Germany; 6 Neuroscience Clinical Research Center, Charité-Universitätsmedizin Berlin, Corporate Member of Freie Universität Berlin and Humboldt Universität zu Berlin, Berlin, Germany; 7 Institute of Biomedicine, School of Medicine, University of Eastern Finland, Kuopio, Finland

**Keywords:** ATAC-seq, chromatin accessibility, immune regulation, integrative transcriptomics, multiple sclerosis, PBMC, RNA-seq, VDR

## Abstract

**Introduction:**

Vitamin D deficiency is a recognized risk factor for multiple sclerosis (MS), yet the *in vivo* molecular mechanisms linking vitamin D3 supplementation to immune regulation remain incompletely understood.

**Methods:**

We performed longitudinal RNA sequencing (RNA-seq) and assay for transposase-accessible chromatin using sequencing (ATAC-seq) profiling of peripheral blood mononuclear cells (PBMCs) from patients with relapsing-remitting MS who either initiated daily high-dose vitamin D3 supplementation or continued regular supplementation, alongside healthy individuals receiving monthly vitamin D3 bolus supplementation. Within-individual changes over the follow-up period were assessed and integrated with published THP-1-derived vitamin D receptor (VDR) binding datasets and previously validated vitamin D target genes.

**Results:**

PBMCs from MS patients retained stable cohort-associated transcriptional and chromatin accessibility signatures that remained distinct from those of the healthy cohort. Vitamin D3 supplementation was not associated with global transcriptomic remodeling but was associated with changes in selected immune-regulatory gene expression programs. In the daily high-dose MS group, candidate genes showing longitudinal expression changes were enriched for innate immune sensing, Toll-like receptor signaling, cytokine-mediated communication, interferon-associated responses, and immune-cell differentiation. Candidate gene sets showed limited overlap across cohorts, although a subset of immune-regulatory and established vitamin D target genes was reproducibly identified. Chromatin accessibility profiling revealed thousands of cohort-specific differentially accessible regions, whereas supplementation-associated accessibility changes were comparatively modest. Integrative analysis prioritized a small set of candidate genes supported by multiple independent datasets, including *AZI2, ABCA2*, and *SIGLEC6*.

**Discussion:**

The findings suggest selective, context-dependent candidate molecular responses to vitamin D_3_ supplementation rather than global immune reprogramming. They also demonstrate the value of integrating transcriptomic and chromatin accessibility data to prioritize vitamin D-responsive pathways and genes for mechanistic validation in larger prospective and randomized studies.

## Introduction

The immune system is regulated by a complex interplay between signals from environment and metabolism, and transcriptional control mechanisms (1, 2). Among these, vitamin D_3_ has emerged as a key immunomodulatory factor that links external stimuli, such as ultraviolet-B exposure, to transcriptional and chromatin-associated responses in immune cells (3). The biologically active metabolite 1α,25-dihydroxyvitamin D_3_ (1,25(OH)_2_D_3_) exerts its effects primarily through the vitamin D receptor (VDR), a ligand-activated transcription factor expressed across innate and adaptive immune cell populations (4, 5). Upon activation, VDR binds to regulatory regions of the genome and modulates chromatin accessibility and transcriptional activity in a highly context-dependent manner (6). This positions vitamin D signaling as an integral component of immune cell plasticity and functional adaptation (7).

Accumulating evidence from *in vitro* studies and experimental models demonstrates that vitamin D influences multiple layers of immune regulation, including antigen presentation, cytokine production, and the differentiation of T cell subsets (8–12). These effects are mediated not only through direct transcriptional control (13) but also through epigenetic mechanisms, such as changes in chromatin accessibility and enhancer activity (6). However, despite detailed mechanistic insights from controlled systems, the extent to which vitamin D induces coordinated transcriptional and chromatin accessibility changes in human immune cells *in vivo* remains incompletely understood. In particular, it is unclear whether vitamin D elicits uniform responses across individuals or instead is linked to heterogeneous, context-dependent immune responses shaped by baseline immune states and environmental factors.

PBMCs provide a tractable system to study systemic immune regulation in humans, capturing the combined responses of multiple immune cell types (14). Recent advances in high-throughput sequencing technologies, including RNA-seq and ATAC-seq, enable the simultaneous characterization of transcriptional activity and its underlying regulatory chromatin landscape. Integration of these approaches provides a framework to characterize transcriptional and chromatin accessibility changes associated with environmental signals *in vivo* (15).

MS is a chronic immune-mediated disease characterized by dysregulated interactions between innate and adaptive immune cells (16). Epidemiological studies have consistently identified vitamin D deficiency as a risk factor for MS susceptibility and disease activity (17–19), yet the molecular mechanisms linking vitamin D status to immune dysregulation in patients remain poorly defined. While clinical trials of vitamin D_3_ supplementation have yielded inconclusive results (20, 21), they have not addressed how distinct supplementation regimens influence immune responses at the transcriptional and chromatin levels. Importantly, in real-world settings, patients frequently adopt heterogeneous vitamin D_3_ supplementation strategies, including high-dose regimens (22, 23), providing an opportunity to investigate context-dependent immune responses under physiologically relevant conditions. We investigated whether (i) distinct vitamin D_3_ supplementation regimens are associated with transcriptional and chromatin accessibility responses that may differ across biological contexts, and (ii) integrative analysis of RNA-seq and ATAC-seq data would identify candidate regulatory pathways linked to immune modulation in MS.

To test this hypothesis, we performed longitudinal multi-omics profiling of PBMCs from individuals with relapsing-remitting MS (RRMS) and healthy controls undergoing different vitamin D_3_ supplementation regimens. By integrating transcriptomic and chromatin accessibility data, we aimed to characterize molecular signatures associated with vitamin D_3_ exposure and to determine whether distinct supplementation strategies are linked to specific immune-associated regulatory responses. Our findings suggest that vitamin D_3_ supplementation is associated with changes in selected immune-related transcriptional and chromatin accessibility programs.

## Materials and methods

### MS cohort

The study cohort comprised 17 patients with RRMS selected as a predefined subgroup from a larger prospective observational MS cohort (n ≈ 200) enrolled in the BERLimmun study (24) (**Table 1**). All participants fulfilled the 2017 McDonald diagnostic criteria for RRMS (25), were between 28 and 55 years of age, had an expanded disability status scale (EDSS) score ≤ 4.0, and had not experienced a clinical relapse within three months prior to baseline assessment. Participants who had changed disease-modifying therapy within the six months before study inclusion were excluded (**Table 2**). No participant changed disease-modifying therapy during the longitudinal follow-up period. At baseline (day 0), all 17 patients underwent standardized clinical assessment and biospecimen collection prior to any modification of vitamin D_3_ supplementation. Following baseline sampling, ten patients voluntarily initiated daily high-dose vitamin D_3_ supplementation, ranging from 20,000 to 70,000 IU/day, at their own risk and under supervision of their personal general practitioner. The remaining seven patients continued regular vitamin D_3_ supplementation (1,000 IU daily or 20,000 IU weekly), with one patient not supplementing at all. Given self-selection and the lack of randomization, we interpret regimen-associated differences descriptively rather than causally. The sample size was determined by the availability of participants within the BERLimmun cohort and was not based on formal power calculations. The study is therefore considered exploratory and hypothesis-generating.

**Table 1:** Participant characteristics, vitamin D_3_ supplementation regimens, and serum 25(OH)D_3_ levels.

Participant	Vitamin D_3_ supplementation	[25(OH)D_3_] in ng/mL at baseline	[25(OH)D_3_] in ng/mL at follow-up	Disease modifying therapy
MS01	60,000 IU/day	40.7	318.0	dimethylfumarat
MS02	30,000 IU/day	66.0	215.0	none
MS03	20,000 IU/day	25.2	60.0	glatirameracetat
MS04	60,000 IU/day	4.3	215.0	none
MS05	60,000 IU/day	48.9	371.0	none
MS06	50,000 IU/day	56.9	267.0	none
MS07	50,000 IU/day	46.9	223.0	none
MS08	70,000 IU/day	40.2	329.0	none
MS09	20,000 IU/day	56.6	144.0	none
MS10	60,000 IU/day	29.2	258.0	dimethylfumarat
MS101	none	25.9	15.0	Interferon beta-1a
MS102	none	24.0	5.8	Ocrelizumab
MS103	20,000 IU/week	40.8	36.8	Ofatumumab
MS104	20,000 IU/week	48.5	54.8	Ocrelizumab
MS105	none	31.0	26.0	none
MS106	20,000 IU/week	50.4	49.7	glatirameracetat
MS107	20,000 IU/week	35.0	28.4	dimethylfumarat
Ind01	60,000 IU/month	26.9	30.7	NA
Ind02	66,500 IU/month	19.3	27.9	NA
Ind03	60,000 IU/month	16.1	20.7	NA
Ind04	60,000 IU/month	38.3	45.9	NA
Ind05	52,000 IU/month	29.5	41.4	NA
Ind06	54,000 IU/month	24.7	32.8	NA
Ind07	66,000 IU/month	21.8	30.8	NA
Ind08	59,000 IU/month	19.9	32.8	NA
Ind09	58,000 IU/month	26.6	37.0	NA
Ind10	67,000 IU/month	18.8	34.9	NA
Ind11	58,000 IU/month	21.5	29.1	NA
Ind12	57,000 IU/month	34.3	27.6	NA
Ind13	55,000 IU/month	47.0	29.8	NA
Ind14	65,000 IU/month	35.3	35.5	NA
Ind16	62,000 IU/month	31.2	31.2	NA
Ind17	54,000 IU/month	27.7	30.3	NA
Ind18	64,500 IU/month	16.8	24.2	NA
Ind19	67,000 IU/month	28.1	33.7	NA
Ind20	69,000 IU/month	20.9	22.2	NA
Ind21	63,500 IU/month	54.8	51.0	NA
Ind23	68,000 IU/month	21.4	19.4	NA
Ind24	85,000 IU/month	41.6	35.8	NA
Ind25	74,000 IU/month	12.7	29.5	NA
Ind26	110,000 IU/month	30.0	32.1	NA
Ind27	92,000 IU/month	41.2	36.7	NA
Ind28	60,000 IU/month	24.5	32.7	NA
Ind29	75,000 IU/month	23.1	22.9	NA
Ind30	85,000 IU/month	15.9	17.8	NA
Ind31	89,000 IU/month	14.3	21.4	NA
Ind32	98,000 IU/month	16.3	24.1	NA
Ind34	77,000 IU/month	33.7	27.4	NA
Ind35	62,000 IU/month	25.7	25.9	NA
Ind36	72,000 IU/month	25.6	28.6	NA
Ind37	64,000 IU/month	15.3	26.1	NA
Ind38	105,000 IU/month	32.4	33.2	NA
Ind39	86,000 IU/month	41.7	26.1	NA
Ind40	93,000 IU/month	26.6	31.2	NA
Ind41	63,000 IU/month	24.9	29.0	NA
Ind42	61,000 IU/month	9.0	18.3	NA
Ind43	80,000 IU/month	17.4	20.7	NA
Ind44	97,500 IU/month	11.4	24.9	NA
Ind45	86,000 IU/month	28.6	31.7	NA

The table summarizes demographic information, vitamin D_3_ supplementation protocols, and serum 25(OH)D_3_ concentrations at baseline (day 0) and at follow-up (day 84 in the healthy cohort; 129 ± 29 days in the MS cohort). MS patients (MS01-MS107) self-initiated either daily high-dose vitamin D_3_, weekly supplementation, low-dose daily supplementation, or no supplementation, while healthy individuals (Ind01-Ind45) received a monthly vitamin D_3_ bolus supplementation adapted to their body mass (1,000 IU/kg). The age range of the MS patients was 28–54 years and that of the healthy individuals 24–64 years.

**Table 2:** Baseline demographic, clinical, imaging, and biochemical characteristics of MS patients by vitamin D_3_ supplementation regimen.

Parameter	Daily high-dose (n = 10)	Regular (n = 7)
Age in years, mean (SD)	46.5 (8.17)	44.7 (10.05)
Female, n (%)	5 (50.0%)	3 (42.9%)
Education in years, mean (SD)	16.2 (1.55)	14.8 (3.36)
BMI in kg/m², mean (SD)	25.6 (5.59)	25.5 (4.92)
EDSS, median [IQR]	2.0 [1.5, 2.9]	1.5 [1.2, 2.8]
Pre-ARR (1 year), mean (SD)	0.6 (0.70)	0.3 (0.49)
Pre-ARR (2 years), mean (SD)	0.5 (0.47)	0.3 (0.57)
Disease-modifying therapies at baseline, n (%)	3 (30.0%)	6 (85.7%)
Number of prior disease-modifying therapies, median [IQR]	0.5 [0.0, 1.0]	1.0 [1.0, 2.0]
MRI T2 lesions count, mean (SD)	42.7 (28.88)	33.0 (33.53)
MRI T2 lesion volume in mm^3^, mean (SD)	5.1 (5.85)	2.8 (3.11)
25(OH)D3 in ng/mL, mean (SD)	41.5 (18.12)	36.5 (10.46)
Vitamin D dose at baseline in IU/day, mean (SD)	3028.6 (3362.32)	1632.7 (1527.21)
PTH in pg/mL, mean (SD)	18.0 (6.61)	24.9 (5.96)
sNfL in pg/mL, mean (SD)	10.7 (4.06)	6.9 (3.68)
T25FW in seconds, mean (SD)	4.4 (1.09)	4.1 (1.07)
9HPT dominant in seconds, mean (SD)	22.8 (6.91)	21.6 (6.50)
9HPT non-dominant in seconds, mean (SD)	23.5 (6.31)	21.1 (4.42)
SDMT, mean (SD)	55.2 (10.76)	54.0 (14.39)
Time since first symptoms in years, mean (SD)	9.9 (9.23)	8.6 (6.51)
Time since diagnosis in years, mean (SD)	5.3 (6.75)	6.9 (6.21)

The table summarizes baseline characteristics of MS patients receiving a daily high-dose vitamin D_3_ (n = 10) or regular supplementation (n = 7). Data are presented as mean with standard deviation (SD), median with interquartile range (IQR), or number and percentage, as indicated. Baseline supplementation refers to vitamin D intake prior to intervention, as daily high-dose supplementation was initiated after baseline assessment.

All participants were followed longitudinally in accordance with the BERLimmun study protocol (24). The BERLimmun study was approved by the Ethics Committee of the Charité-Universitätsmedizin Berlin (approval number EA1/362/20) and was conducted in accordance with the Declaration of Helsinki and applicable national regulations. All participants provided written informed consent prior to inclusion and before any study-related procedures were performed. The primary exposure variable was the vitamin D_3_ supplementation regimen. Primary outcome variables included transcriptomic changes assessed by RNA-seq and chromatin accessibility changes assessed by ATAC-seq. Covariates included demographic characteristics, clinical parameters, and baseline vitamin D status. The study is reported in accordance with the STROBE guidelines for observational studies.

### Cohort of healthy individuals

Healthy individuals were recruited within the VitDPAS vitamin D intervention study, which commenced in November 2023 in Olsztyn, Poland (26). Extensive exclusion criteria were applied to ensure the absence of acute or chronic conditions that could interfere with vitamin D metabolism, calcium homeostasis, immune function, or study procedures. These included self-reported recent infections; gastrointestinal, renal, hepatic, endocrine, neurological, oncological, or inflammatory diseases; disorders affecting calcium metabolism; use of medications known to alter 25-hydroxyvitamin D_3_ (25(OH)D_3_) levels; substance abuse; mental illness; pregnancy or breastfeeding; and participation in interventional studies within the preceding four months. The final cohort comprised 42 healthy participants (21 females and 21 males), aged 24–64 years with a body mass index (BMI) ranging from 19.3 to 29.3 kg/m^2^ (**Table 1**). Participants received a single oral vitamin D_3_ bolus of 1,000 IU per kilogram body weight every 28 days, administered with breakfast. This dosing regimen corresponds to a monthly vitamin D_3_ intake and falls within established safety margins, comparable to dosing strategies used in previous long-term vitamin D_3_ intervention studies (27). The resulting dose range of 52,000-110,000 IU vitamin D_3_ per month, corresponding to an average daily intake of approximately 1,700-3,700 IU/day, was therefore considered safe. The VitDPAS study protocol was approved by the Ethics Committee of the Olsztyn Chamber of Doctors (approval number 31/2023/VIII). The study was conducted in accordance with relevant ethical guidelines and regulations, and all participants provided written informed consent prior to participation.

### PBMC isolation

Blood samples were collected immediately before the first supplementation (day 0) and after 84 days (follow-up), corresponding to three consecutive bolus administrations every 4 weeks, for the VitDPAS cohort. The second visit of the MS patients occurred approximately 3–5 months (129 ± 29 days) after baseline, with the exact interval varying between participants. For simplicity and consistency with the VitDPAS cohort, this follow-up time point is referred to throughout the manuscript as the follow-up time point (day 84 in the VitDPAS cohort; 129 ± 29 days in the MS cohort). Serum 25(OH)D_3_ concentrations were measured using an electrochemiluminescence binding assay on a Cobas Pure Immunoassay Analyzer (Roche) at the analytical laboratories of the Municipal Polyclinical Hospital in Olsztyn, Poland, and Charité-Universitätsmedizin Berlin, Germany.

PBMCs from patients with MS were isolated from heparinized whole blood by density gradient centrifugation. Blood samples were diluted in phosphate-buffered saline (PBS; without Ca^2+^/Mg^2+^), layered over Ficoll, and centrifuged without braking. The PBMC layer at the plasma-Ficoll interface was collected, washed twice with cold PBS and once with RPMI-based wash medium, and centrifuged at 4 °C. Cell number and viability were assessed by Trypan Blue exclusion using a hemocytometer. PBMCs were cryopreserved in RPMI supplemented with fetal calf serum and DMSO using controlled-rate freezing prior to long-term storage in liquid nitrogen. PBMCs from the 42 participants of the VitDPAS study were isolated from 8 mL of peripheral blood collected in Vacutainer CPT Cell Preparation Tubes containing sodium citrate (Becton Dickinson). Following isolation, cells were washed with PBS, aliquoted at a concentration of 4 × 10^6^ cells/mL, and stored at −80 °C.

### Transcriptome analysis

For all cohorts total RNA was extracted from PBMCs using the High Pure RNA Isolation Kit (Roche) according to the manufacturer’s instructions. RNA integrity and quality were assessed using an Agilent TapeStation. Following ribosomal RNA depletion, sequencing libraries were prepared using kits and protocols from New England Biolabs. RNA-seq libraries were sequenced with a read length of 75 bp on an Illumina NextSeq 2000 platform at the EMBL Gene Core (Heidelberg, Germany). Sequencing quality was evaluated using FastQC (v0.12.1; **Supplementary Table 1**). Reads were aligned to the human reference genome GRCh38.p14 (Ensembl release 114) using STAR (v2.7.10b) (28), and gene-level quantification was performed with featureCounts (v2.0.6) (29) using default parameters. Gene nomenclature was standardized to Human Gene Nomenclature Committee (HGNC) symbols using HGNChelper (v0.8.1). Gene annotations, including identifiers, descriptions, genomic coordinates, and biotypes, were obtained from Ensembl (release 111) via biomaRt (2.58.2) (30). Entrez Gene identifiers were added using org.Hs.eg.db (3.18.0), with missing mappings manually verified through NCBI. Genes lacking genomic position information or encoded by mitochondrial DNA were excluded, and analyses were restricted to protein-coding genes.

### Differential gene expression analysis

Differential gene expression analysis was conducted in R (version 4.3.1) on macOS 14.0 using the edgeR package (version 4.0.16) (31). Analyses were restricted to 19,238 protein-coding genes in order to reduce transcriptional noise associated with lowly expressed or non-coding transcripts. Raw read counts were normalized to counts per million (CPM) to account for differences in library size. Low-expression genes were filtered using the filterByExpr() function in edgeR, thereby reducing the multiple-testing burden and improving statistical robustness. Following filtering, library sizes were recalculated and normalized using the trimmed mean of M-values (TMM) method.

Multidimensional scaling (MDS) analysis was performed on normalized log-CPM values using the plotMDS() function in edgeR with default variance-based gene selection. In this representation, distances between samples approximate the typical log_2_ fold change (log_2_FC) between expression profiles and were calculated as the root mean square deviation (Euclidean distance) of log_2_FC values across genes contributing most strongly to sample separation. Volcano plots were generated in R using the ggplot2 package, with log_2_FC values plotted against -log_10_p-values.

Differential expression analysis was conducted within the generalized linear model quasi-likelihood (GLM-QL) framework implemented in edgeR using raw read count data. The longitudinal paired design enabled within-individual comparisons between baseline and follow-up samples, thereby reducing inter-individual variability. Negative binomial dispersions were estimated using the Cox-Reid profile-adjusted likelihood method with robust empirical Bayes shrinkage to minimize the influence of outlier genes. Gene-wise models were fitted using glmQLFit(), and statistical significance was assessed using quasi-likelihood F-tests implemented in glmQLFTest().

Differential expression was initially assessed using FDR-adjusted p-values. In the longitudinal MS supplementation analyses, no genes reached transcriptome-wide significance after multiple-testing correction (FDR < 0.05). Therefore, exploratory downstream analyses focused on candidate genes fulfilling predefined thresholds of nominal p-value < 0.05, absolute log_2_FC > 0.25, and log_2_CPM > 1 (**Supplementary Table 2**). These candidate-gene sets were used for hypothesis-generating analyses, including pathway enrichment and integrative multi-omics prioritization. Statistical analyses were performed separately within each cohort and supplementation regimen using paired within-individual comparisons. Participant identity was incorporated into the design matrix to account for subject-specific variability. Because of the limited sample size and observational study design, no formal adjustment for additional covariates was performed. Consequently, comparisons between MS patients and healthy individuals were interpreted descriptively and used primarily to identify shared or context-dependent regulatory patterns rather than direct quantitative contrasts.

Enrichment analyses were performed using the EnrichR webtool (32, 33) with the supplementation-associated candidate-gene sets defined by nominal p-value < 0.05, absolute log_2_FC > 0.25, and log_2_CPM > 1. Upregulated and downregulated genes were analyzed together because the aim was to identify biological processes represented within the candidate-gene sets rather than to infer pathway activation states. Pathway significance was assessed using the multiple-testing-corrected statistics reported by EnrichR.

### Cross-cohort integration and transcriptome visualization analyses

To integrate gene expression data from the MS and VitDPAS cohorts, common gene identifiers were matched across both datasets, and only genes present in both cohorts were retained for downstream analyses. All samples were processed and sequenced in the same laboratory using the same experimental protocols, and raw read counts from both cohorts were normalized together to CPM to account for differences in sequencing depth. Because the MS and VitDPAS cohorts differ in disease status, age distribution, baseline vitamin D levels, and supplementation regimens, cross-cohort comparisons are interpreted cautiously and serve primarily to identify shared or context-specific regulatory responses rather than to support direct quantitative comparisons. Given the limited sample size and observational design, no formal adjustment for potential confounders was performed. Analyses focused on within-individual comparisons and descriptive identification of consistent regulatory patterns.

RNA-seq heatmaps were generated in R using the pheatmap package. These heatmaps were used for visualization of expression patterns within predefined gene sets and were not intended as independent statistical evidence of cohort separation. Conclusions regarding transcriptomic differences between cohorts are based primarily on unsupervised analyses of the complete transcriptome, including hierarchical clustering and MDS analyses. CPM-normalized expression matrices were used for downstream visualization analyses. For heatmap generation, expression values were additionally normalized on a per-gene basis using min-max normalization according to the formula:


x_norm = (x−min(x))/(max(x)−min(x))

This transformation scaled each gene between 0 and 1 across samples to facilitate visualization of relative expression patterns independent of absolute expression magnitude. Hierarchical clustering of samples was performed using Euclidean distance and complete linkage clustering for whole-transcriptome heatmaps. For selected analyses focusing on MS-related genes, *k-means* clustering (k = 2) was additionally applied to identify broad expression patterns distinguishing healthy individuals and MS patients. Heatmaps were visualized using a blue-white-red color scale, where blue represented lower relative expression and red represented higher relative expression.

### Immune cell deconvolution analysis

To estimate potential changes in PBMC composition following vitamin D_3_ supplementation, transcriptome-based immune cell deconvolution was performed using CIBERSORTx (34). Normalized RNA-seq expression data (**Supplementary Table 2**) were analyzed using reference immune-cell signatures to estimate the relative abundance of major PBMC populations. Comparative analyses focused on monocytes, T cells, B cells, and natural killer (NK) cells at baseline (day 0) and follow-up (day 84) across all supplementation groups. The analysis was intended to assess whether large-scale shifts in major immune-cell populations could account for observed transcriptional differences.

### ATAC-seq

For ATAC-seq, nuclei were isolated from 100,000 PBMCs by incubation in 50 µL of lysis buffer (10 mM Tris-HCl, pH 8.0; 10 mM NaCl; 3 mM MgCl_2_; 0.1% Tween-20; 0.1% IGEPAL CA-630; 0.01% digitonin). After 3 minutes on ice, 1 mL of wash buffer (10 mM Tris-HCl, pH 8.0; 10 mM NaCl; 3 mM MgCl_2_; 0.1% Tween-20) was added. Nuclei were pelleted and subjected to transposition using a Tn5 transposase reaction mix (Illumina) supplemented with 0.01% digitonin and 0.1% Tween-20. The transposition reaction was performed at 37 °C for 30 minutes in a thermomixer. Transposed DNA was purified using the DNA Clean & Concentrator-5 Kit (Zymo Research), followed by PCR amplification, purification, and size selection using SPRISelect beads (Beckman Coulter). Library quality was assessed with an Agilent TapeStation. Indexed libraries were quantified, pooled, and prepared for sequencing. ATAC-seq libraries were sequenced using 75 bp paired-end reads on an Illumina NextSeq 2000 platform at the EMBL GeneCore Facility (Heidelberg, Germany), following standard protocols. Baseline and follow-up samples from the different study groups were processed using the same library-preparation workflow. Samples from different cohorts, supplementation groups, and time points were randomized across library-preparation and sequencing batches and distributed across sequencing runs to minimize potential confounding between batch effects and biological variables.

### Epigenome analysis

Quality control of raw sequencing data was performed using FastQC (v0.12.1). Adapter sequences were trimmed with Trim Galore (v0.6.10). Paired-end reads were aligned to the human reference genome GRCh38.p14 using STAR (v2.7.10b) with default parameters unless otherwise specified. STAR was used to ensure methodological consistency with RNA-seq alignment pipelines. Read mapping statistics are summarized in **Supplementary Table 1**. Reads mapping to ENCODE blacklisted regions or exhibiting low mapping quality (MAPQ < 2) were removed using the samtools view function with the -q 2 option. PCR duplicates were eliminated using samtools markdup (v1.9) to minimize amplification bias. Peak calling was performed using MACS3 (v3.0.1) with the callpeak function and the following parameters: -f BAMPE –verbose 3 –shift 0 -g hs -q 0.01 -B. Peak overlap analyses were conducted using bedtools (v2.30.0) and bedops (v2.4.41). Heatmaps of chromatin accessibility were produced with the plotHeatmap function from deepTools (v3.5.6) (35), using genomic coordinates of significant peaks identified in the consensus ATAC-seq dataset.

### Differential chromatin accessibility analysis

Differential chromatin accessibility analysis was performed in R (v4.4.0) on macOS 15.7.3 using the DiffBind package (v3.14.0) (31). A consensus peak set was generated with the dba.count function using a summit width of 75 bp, followed by merging of overlapping summits. This procedure resulted in a consensus set of 28,105 peak summits (**Supplementary Table 3**).

Peak annotation was carried out using the ChIPseeker package (v1.40.0) in combination with the TxDb.Hsapiens.UCSC.hg38.knownGene annotation database (v3.18.0) and org.Hs.eg.db (v3.19.1). Differential chromatin accessibility was assessed through pairwise comparisons between follow-up versus baseline samples using DESeq2-based statistical methods implemented within the DiffBind framework. Analyses were performed separately within each cohort and supplementation regimen using the paired longitudinal study design.

Genomic regions were classified as differentially accessible at a nominal p-value < 0.05, which was treated as an exploratory threshold. Interpretation emphasized consistency across biological patterns and integration with transcriptomic and external regulatory datasets rather than isolated peak-level significance. Because stringent quality-control filtering substantially reduced the number of paired MS samples available for ATAC-seq analysis, statistical power for differential accessibility testing was limited.

Although both RNA-seq and ATAC-seq analyses included nominal p-value-based assessments, transcriptomic analyses generally showed stronger statistical support and greater reproducibility across comparisons. In contrast, chromatin accessibility analyses were interpreted more conservatively and prioritized broader biological consistency, overlap with transcriptional signatures, and convergence with published VDR-associated regulatory datasets (36, 37) rather than reliance on individual differentially accessible loci.

Potential sources of bias include self-selection into supplementation regimens, differences in baseline vitamin D status, and the observational study design. In addition, PBMCs represent a heterogeneous mixture of immune-cell populations, thereby limiting resolution of cell-type-specific chromatin accessibility changes. Differences between MS patients and healthy individuals in age, disease status, and supplementation protocols further constrain direct cross-cohort comparability. Because these factors were not experimentally controlled, the findings should be interpreted as descriptive associations rather than evidence of causal regulatory effects.

ATAC-seq heatmaps were generated in R using normalized chromatin accessibility matrices derived from DiffBind analyses. Consensus chromatin accessibility profiles were visualized across samples to assess global regulatory patterns. For selected analyses, *k-means* clustering (k = 2) was applied to accessibility profiles in order to identify broad chromatin accessibility patterns separating healthy individuals from MS patients. Heatmaps were displayed using a blue-white-red color scale, where blue indicates lower relative chromatin accessibility and red indicates higher relative chromatin accessibility across samples. Motif enrichment analysis was performed using HOMER (38) on supplementation-associated differentially accessible chromatin regions identified in the high-dose MS group. Motifs were considered significant when both nominal p-values and Benjamini-Hochberg adjusted q-values were < 0.05.

Because the study was exploratory and included a limited number of paired longitudinal samples, particularly for ATAC-seq analyses, nominal significance thresholds were used to prioritize biologically coherent regulatory patterns for integrative downstream analyses rather than to establish definitive locus-specific effects.

## Results

### Study cohorts and effects of vitamin D_3_ supplementation on biochemical and clinical parameters

**Figure 1:**
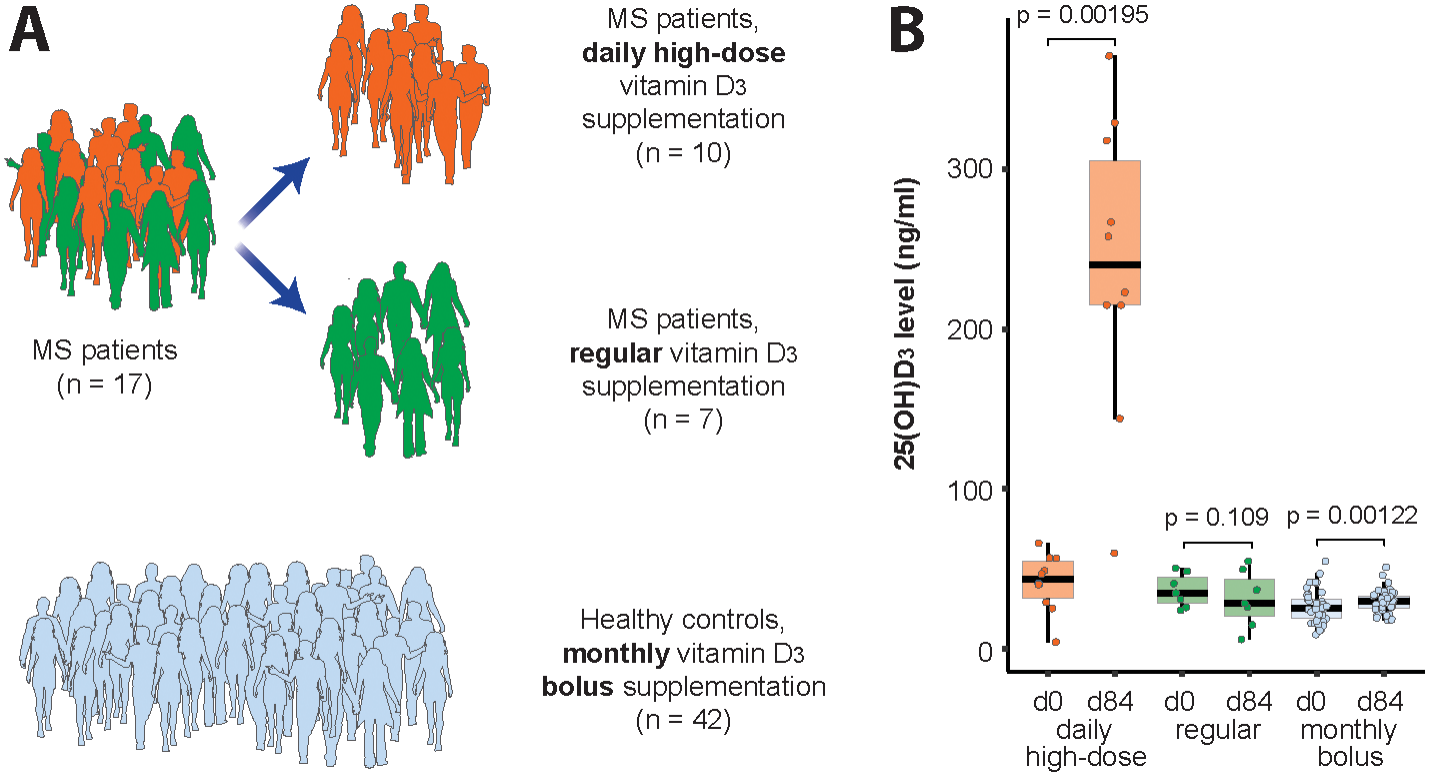
Study design and serum 25(OH)D_3_ responses to vitamin D_3_ supplementation. Overview of the study cohorts **(A)**. Seventeen MS patients were stratified into a daily high-dose vitamin D_3_ supplementation group (n = 10) and a regular vitamin D_3_ supplementation group (n = 7). Healthy individuals received monthly vitamin D_3_ bolus supplementation (n = 42). Serum 25(OH)D_3_ concentrations at baseline (day 0) and follow-up (129 ± 29 days in MS patients and 84 days in healthy individuals) **(B)**. Boxplots show median and interquartile range; points represent individual participants. Statistical significance was assessed using paired Wilcoxon signed-rank tests comparing baseline and follow-up samples within each cohort.

We analyzed 17 relapsing-remitting MS patients from the BERLimmun study (24), dividing them post-baseline into high-dose (n = 10) and regular-supplementation (n = 7) groups (**Figure 1A**). In the high-dose group, the patients self-initiated vitamin D_3_ supplementation, ranging from 20,000 to 70,000 IU/day, under general practitioner supervision (**Table 1**). Four of the remaining seven patients continued regular vitamin D_3_ supplementation (1,000 IU daily or 20,000 IU weekly), with three patients not supplementing at all. At baseline (day 0), the two MS groups showed similar distributions of age, disease duration, EDSS score, and serum 25(OH)D_3_ concentrations (**Table 2**). However, because of the observational design and heterogeneous treatment exposure, the groups should not be considered fully matched.

A control cohort of 42 healthy individuals belonging to the VitDPAS study (26) received over three months vitamin D_3_ supplementation every 28 days at a dose of 1,000 IU per kilogram body weight (**Table 1**). This group was younger on average (39.9 years) and exhibited lower baseline serum 25(OH)D_3_ levels (26.0 ng/mL) compared with both MS groups. Accordingly, cross-cohort comparisons were interpreted as contextual rather than strictly matched analyses.

For serum collection and PBMC isolation, blood samples were obtained at baseline and follow-up. The follow-up visit occurred after 129 ± 29 days in MS patients and after 84 days in healthy individuals. Daily high-dose vitamin D_3_ supplementation in MS patients resulted in a significant increase in serum 25(OH)D_3_ concentrations to a mean of 240 ng/mL (p = 0.00195; **Figure 1B**). No clinical symptoms suggestive of vitamin D toxicity were reported during the observation period. However, the study was not designed as a formal safety assessment, and systematic monitoring of hypercalcemia, renal-function parameters, or other toxicity markers was not performed. Accordingly, the absence of reported adverse events should not be interpreted as evidence for the safety of the achieved serum 25(OH)D_3_ concentrations. The increase in circulating 25(OH)D_3_ concentrations was accompanied by a corresponding reduction in parathyroid hormone (PTH) levels (**Table 3**). In contrast, regular supplementation in the MS control group did not significantly alter serum 25(OH)D_3_ concentrations. Healthy individuals receiving monthly vitamin D_3_ bolus supplementation exhibited a more modest but still significant increase in serum 25(OH)D_3_ levels to 29.6 ng/mL (p = 0.00122).

**Table 3:** Clinical, biochemical, and functional outcomes in MS patients receiving daily high-dose versus regular vitamin D_3_ supplementation.

Outcome	Daily high-dose (Mean ± SD/Median [IQR])	Regular (Mean ± SD/Median [IQR])	P-value
EDSS (score)	-0.5 [-0.5 – 0.8]	-0.5 [-0.8 – 0.0]	0.65
25(OH)D3 in ng/mL	+210.4 [155.8 – 265.2]	-5.0 [-8.8 – -2.3]	<0.001
Vitamin D3 dose in IU/day	+52000.0 [26071.4 – 60000.0]	+0.0 [0.0 – 0.0]	<0.001
PTH in pg/mL	-4.2 [-5.8 – 0.2]	+4.0 [-4.2 – 8.6]	0.16
SDMT (score)	-0.5 [-2.5 – 2.8]	+1.0 [-0.5 – 5.0]	0.35
T25FW in seconds	+0.2 [-0.1 – 0.7]	+0.4 [-0.1 – 0.6]	0.88
9HPT dominant hand in seconds	-0.2 [-2.7 – 1.2]	+0.0 [-0.4 – 0.6]	0.74
9HPT non-dominant hand in seconds	+0.2 [-1.1 – 1.4]	+0.3 [-1.6 – 1.7]	0.67
EDSS progression within 3 months, n (%)	2 (20.0%)	0 (0.0%)	–
New relapses within 3 months, n (%)	2 (20.0%)	0 (0.0%)	–
Follow-up interval in months, median [IQR]	4.6 [4.0, 5.2]	3.9 [3.4, 5.1]	–

Apart from supplementation-associated differences in vitamin D status, no relevant longitudinal changes were observed in either MS group with respect to EDSS scores, serum neurofilament light chain (sNfL) levels, or functional performance measures, including the timed 25-foot walk (T25FW), 9-hole peg test (9HPT), and symbol digit modalities test (SDMT) (**Table 3**).

Collectively, daily high-dose vitamin D_3_ supplementation over three months in MS patients produced a markedly distinct systemic vitamin D exposure trajectory compared with regular-dose MS patients and healthy controls, resulting in substantially elevated circulating 25(OH)D_3_ concentrations and reduced PTH levels. Despite these pronounced biochemical differences, clinical, biomarker, and functional outcome measures remained largely unchanged over the observation period.

### Distinct baseline PBMC transcriptomic profiles distinguish the MS and healthy cohorts

PBMCs were collected from 17 MS patients and 42 healthy individuals at baseline and follow-up. Total RNA was isolated and subjected to RNA-seq analysis. Hierarchical clustering of all 19,238 protein-coding genes suggested, at both time points, a clear separation between the transcriptomes of MS patients and healthy individuals (**Supplementary Figure 1**). This separation was observed using the complete transcriptome and therefore is independent of subsequent feature-selection procedures used for visualization. Focusing on samples from baseline (day 0), ten major gene clusters could be identified (**Supplementary Table 2**), three of which (clusters #8, #9, and #10) comprised a total of 6,197 genes that were not or only very weakly expressed (CPM < 1). Differential gene expression analysis of the remaining 13,041 genes comparing MS patients and healthy individuals identified 1,653 significantly differentially expressed genes (FDR < 0.05) at baseline and 1,569 genes at follow-up, with 1,480 genes overlapping between both time points (**Supplementary Table 2**).

**Figure 2:**
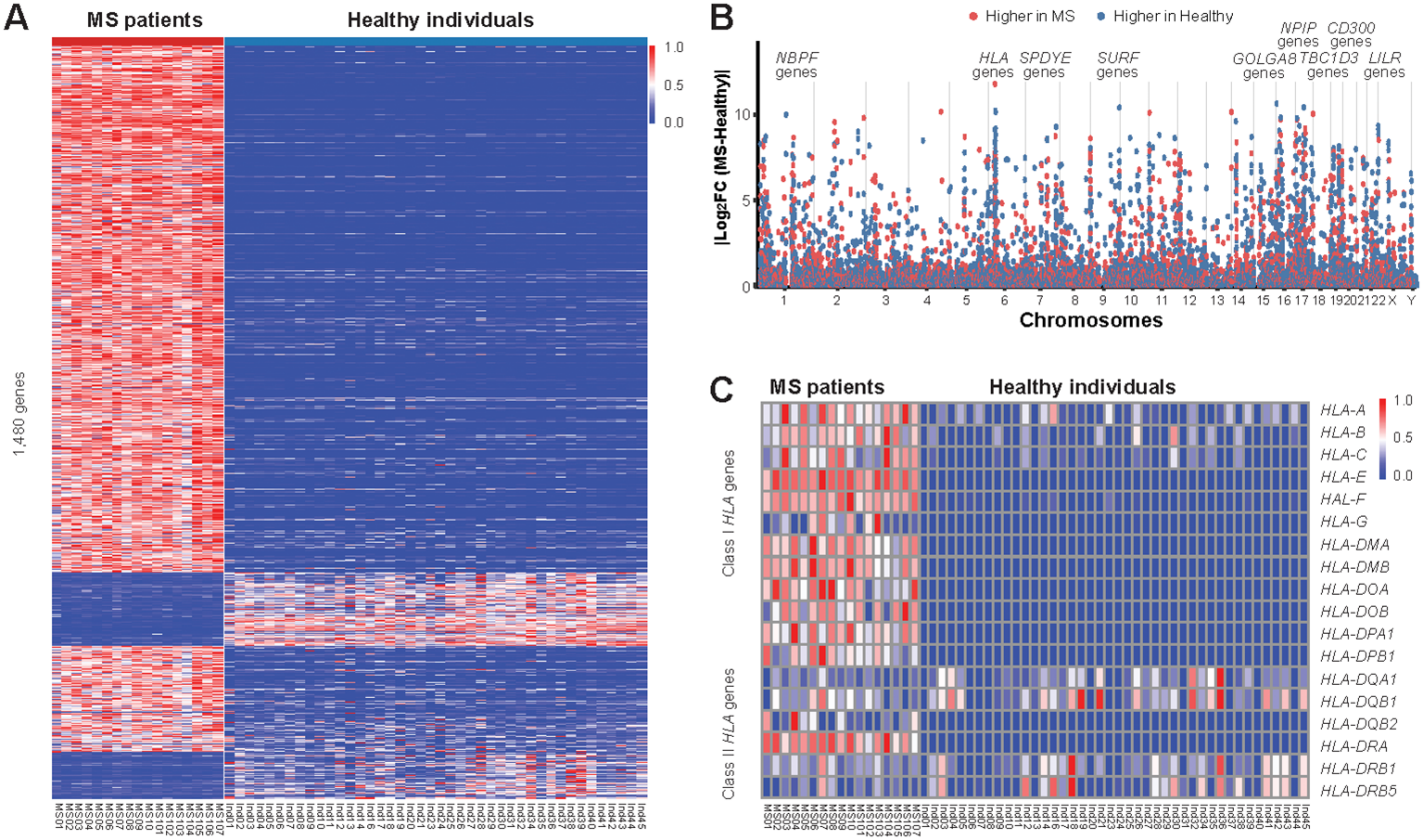
Baseline transcriptomic differences between MS patients and healthy individuals. Heatmap of 1,480 genes differentially expressed between MS patients (n = 17) and healthy individuals (n = 42) at baseline, identified by RNA-seq analysis using edgeR (FDR < 0.05) **(A)**. Expression values represent CPM-normalized counts subjected to per-gene min-max normalization (0–1 scaling). Samples were grouped using *k-means* clustering (k = 2), and genes were hierarchically clustered using Euclidean distance and complete linkage. Genomic distribution of the 1,480 differentially expressed genes across human chromosomes **(B)**. Red and blue dots indicate genes expressed at higher levels in MS patients and healthy individuals, respectively. Heatmap of differentially expressed *HLA* genes identified at baseline. Expression values were normalized as in panel A **(C)**.

For the majority (84.2%) of the shared genes, expression levels were higher in MS patients than in healthy individuals (**Figure 2A**). A Manhattan plot highlighted increased expression of genes located in genomic regions containing genes involved in antigen presentation and immunoregulatory receptor signaling such as *NBPF* (NBPF member, chromosome 1), *HLA* (major histocompatibility complex, chromosome 6), *SPDYE* (speedy/RINGO cell cycle regulator family member, chromosome 7), *SURF* (surfeit, chromosome 9), *GOLGA8* (golgin A8 family member, chromosome 15), *NPIP* (nuclear pore complex interacting protein family member, chromosome 16), *TBC1D3* (TBC1 domain family member, chromosome 17), *CD300* (CD300 molecule like family member, chromosome 17) and *LILR* (leukocyte immunoglobulin like receptor, chromosome 19) (**Figure 2B**).

Among these, the *HLA* locus represents the strongest established genetic susceptibility region for MS and therefore provides biological plausibility for the observed cohort differences (39) (**Figure 2C**). In most MS patients, all members of the *HLA* class I family and the majority of *HLA* class II genes were expressed. In contrast, healthy individuals exhibited higher expression of the *HLA* class II genes *HLA-DQA1*, *HLA-DQB1*, *HLA-DRB1*, and *HLA-DRB5*, which were comparatively less expressed in immune cells from MS patients (**Supplementary Figure 2**).

The *CD300* and *LILR* gene families encode immunomodulatory receptors expressed on myeloid and lymphoid cells and therefore represent biologically plausible candidates for further investigation in the context of MS, although they are not established MS susceptibility loci (40, 41). In contrast, the remaining gene families identified in enriched genomic regions have not been previously linked to MS pathogenesis and may reflect broader immune activation states or structural characteristics of segmentally duplicated genomic regions rather than disease-specific genetic associations.

In summary, RNA-seq profiling suggested reproducible transcriptional differences between the MS and healthy cohorts, characterized by differential expression of numerous immune-related genes.

### Comparison of supplementation-associated candidate transcriptomic responses across cohorts

**Figure 3:**
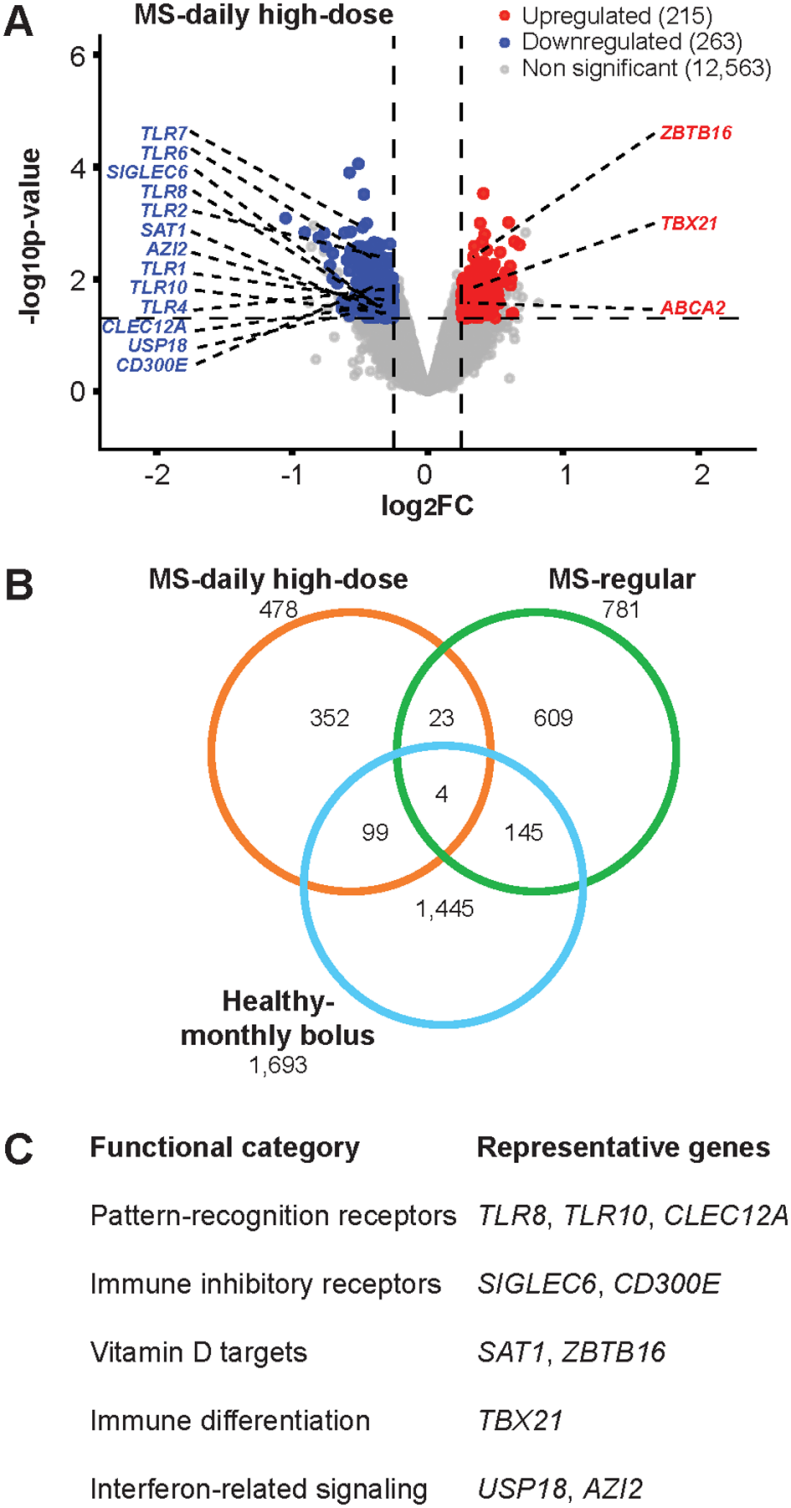
Vitamin D-responsive transcriptional signatures in MS patients and healthy individuals. Volcano plot showing differential gene expression between baseline and follow-up in MS patients receiving daily high-dose vitamin D_3_ supplementation (n = 10) **(A)**. The x-axis indicates log_2_FC and the y-axis –log_10_(p-value). Red and blue dots represent significantly upregulated and downregulated genes, respectively, defined by nominal p < 0.05 and |log_2_FC| > 0.25, whereas gray dots indicate non-significant genes. Selected supplementation-associated candidate genes are highlighted. Numbers of upregulated, downregulated, and non-significant genes are indicated. Venn diagram showing the overlap of candidate supplementation-responsive genes among the three supplementation groups: MS patients receiving daily high-dose vitamin D_3_, MS patients receiving regular supplementation, and healthy individuals receiving monthly vitamin D_3_ bolus supplementation **(B)**. Gene sets were defined using nominal p < 0.05 and |log_2_FC| > 0.25. Numbers indicate unique and shared differentially expressed genes. Functional categories represented among 103 supplementation-associated candidate genes shared between MS patients receiving daily high-dose vitamin D_3_ supplementation and healthy individuals receiving monthly vitamin D_3_ bolus supplementation **(C)**. Selected representative genes are indicated.

Next, we investigated transcriptional responses associated with different vitamin D_3_ supplementation regimens (**Supplementary Table 2**). No genes reached transcriptome-wide significance after FDR correction in the longitudinal MS supplementation analyses. Therefore, exploratory downstream analyses focused on candidate genes identified using predefined thresholds of nominal p-value < 0.05, absolute log_2_FC > 0.25, and log_2_CPM > 1. In the daily high-dose MS group, 478 genes fulfilled these criteria (215 upregulated and 263 downregulated; **Figure 3A**). Corresponding exploratory candidate-gene sets comprised 781 genes in the regular-supplementation MS group (387 upregulated and 394 downregulated; **Supplementary Figure 3A**) and 1,693 genes in healthy individuals receiving monthly vitamin D_3_ bolus supplementation (905 upregulated and 788 downregulated; **Supplementary Figure 3B**). Because sample sizes differed substantially between cohorts, these numbers should not be interpreted as quantitative measures of response magnitude. Moreover, follow-up duration differed between cohorts, with MS patients sampled after an average of 129 ± 29 days and healthy individuals after 84 days. Therefore, differences in candidate-gene responses between cohorts may reflect not only biological context and supplementation regimen but also differences in exposure duration.

To determine whether transcriptional changes reflected major shifts in PBMC composition, we performed transcriptome-based immune-cell deconvolution using CIBERSORTx. Estimated proportions of monocytes, T cells, B cells, and NK cells remained stable between baseline and follow-up in all groups (**Supplementary Figure 4**), arguing against large-scale changes in PBMC composition as the primary driver of the observed transcriptional responses. Consistently, principal component and MDS analyses did not identify global transcriptome-wide shifts after supplementation, whereas analyses restricted to candidate supplementation-associated candidate gene sets demonstrated coherent within-individual expression changes over time (**Supplementary Figure 5**).

Comparison of the three supplementation-associated candidate gene sets identified only limited overlap (**Figure 3B**). Among the 478 genes regulated in the daily high-dose MS group, 103 (21.5%) were also identified in healthy individuals, whereas only 27 (5.6%) overlapped with the regularly supplemented MS group. The limited overlap between threshold-defined candidate-gene sets should be interpreted cautiously because statistical power differed substantially among cohorts. Shared candidate genes included in innate immune sensing and immune regulation, such as *TLR8* (Toll-like receptor 8), *TLR10*, *CD300E*, and *SIGLEC6* (sialic acid binding Ig like lectin 6), as well as the established vitamin D target genes *SAT1* (spermidine/spermine N1-acetyltransferase 1) and *ZBTB16* (zinc finger and BTB domain containing 16) (26). These findings suggest that supplementation-associated candidate transcriptional responses may differ across study cohorts and supplementation contexts, although a subset of immune-regulatory targets is reproducibly detected across cohorts.

To further assess the relationship between systemic vitamin D exposure and transcriptional responsiveness, we correlated changes in serum 25(OH)D_3_ concentrations with gene expression changes in the daily high-dose MS group (**Supplementary Figure 6**). Several genes involved in innate immune signaling, including *CLEC12A* (C-type lectin domain family 12 member A), *CD36* (CD molecule), *JAK2* (janus kinase 2), *SAT1*, *TLR2*, *TLR7*, and *TLR10*, displayed negative correlations with changes in serum vitamin D levels, whereas *USP18* (ubiquitin specific peptidase 18) showed a positive correlation. These observations are consistent with an association between vitamin D status and immune-regulatory gene expression rather than uniform transcriptional activation or repression.

Of the 1,480 genes differentially expressed between MS patients and healthy individuals, 45 were also vitamin D-responsive in the daily high-dose MS group. STRING analysis suggested a sparse interaction network without a dominant hub-centered module (**Supplementary Figure 7**). Nevertheless, several overlapping genes have established roles in immune regulation and MS pathophysiology, including *HLA-DPB1*, *CR1* (complement C3b/C4b receptor 1), *ZAP70* (zeta chain of T cell receptor associated protein kinase 70), *AZI2* (5-azacytidine induced 2), *NLRC4* (NLR family CARD domain containing 4), *LILRB4*, and *PVRIG* (PVR related immunoglobulin domain containing). Together, these findings suggest that supplementation-associated candidate transcriptional responses differed among the analyzed cohorts and supplementation regimens, although the present study was not powered to formally compare longitudinal response magnitudes between groups.

Taken together, these descriptive analyses identify both shared and cohort-specific supplementation-associated candidate responses involving genes related to innate immune sensing, immune regulation, and inflammatory signaling.

### Supplementation-associated candidate genes in the high-dose MS group were enriched for innate immune and inflammatory signaling pathways

A central aim of this study was to explore molecular signatures potentially associated with daily high-dose vitamin D_3_ supplementation in MS patients. Because no genes remained significant after multiple-testing correction, the following analyses are based on exploratory vitamin D-responsive genes and should be interpreted as hypothesis-generating. Gene ontology (GO) analysis of the 478 candidate supplementation-responsive genes identified in the 10 MS patients who self-initiated this regimen suggested significant enrichment of biological processes related to innate immune activation and inflammatory signaling (**Figure 3C**; **Table 4**). Because these analyses were based on exploratory candidate-gene sets rather than transcriptome-wide significant genes, the resulting enrichments should be interpreted as descriptive biological themes rather than definitive pathway-level effects. The most significantly enriched terms included positive regulation of defense response, pattern-recognition receptor signaling, TLR signaling, cytokine-mediated signaling, inflammatory response regulation, TNF production, and IL1β production. These enrichments were driven largely by seven *TLR* genes (*TLR1*, *TLR2*, *TLR4*, *TLR6*, *TLR7*, *TLR8*, and *TLR10*) that responded to vitamin D_3_ supplementation. Importantly, enrichment analysis identifies biological processes represented within the responsive gene set and does not imply uniform activation or repression of these pathways. Consequently, the identified enrichments indicate biological themes represented within the candidate-gene set and should not be interpreted as definitive evidence for pathway activation or repression.

**Table 4:** GO enrichment analysis of MS- and vitamin D-associated genes.

Term	Overlap	Padj	Genes
Positive regulation of defense response (GO:0031349)	17 of 130	1.38x10^-8^	TASL; MEF2C; IL15; TRADD; FPR2; NLRC4; AIM2; CASP5; MDK; CASP1; IFNK; S100A12; TLR8; TLR7; TLR4; CCR2; TLR2
Pattern recognition receptor signaling pathway (GO:0002221)	12 of 65	3.86x10^-8^	FCN1; TLR1; CASP1; TLR8; FFAR2; TLR10; TLR7; TLR6; NLRC4; TLR4; CTSS; TLR2
Positive regulation of response to external stimulus(GO:0032103)	18 of 163	7.53x10^-8^	TASL; IL15; TRADD; CD180; FPR2; NLRC4; SCIMP; AIM2; CASP5; MDK; CASP1; IFNK; S100A12; TLR8; TLR7; TLR4; CCR2; TLR2
TLR signaling pathway (GO:0002224)	9 of 35	9.59x10^-8^	TLR1; TLR8; TLR10; TLR7; TLR6; SCIMP; TLR4; CTSS; TLR2
Positive regulation of cytokine production (GO:0001819)	24 of 325	0.0000012	CD86; FCN1; CLEC12A; IL15; FLT4; GATA3; PRKCZ; SCIMP; C3; TLR1; TMIGD2; MDK; CLEC6A; CASP1; TLR8; FFAR2; TLR7; TLR6; JAK2; ZFPM1; TLR4; TMEM106A; CCR2; TLR2
Cellular response to cytokine stimulus (GO:0071345)	22 of 289	0.0000019	CCR1; STAT1; TRADD; IL31RA; GATA3; LILRB4; CSF2RA; LILRA4; HCK; AIM2; OAS1; RUFY4; CASP1; IFNK; ST3GAL6; MNDA; JAK2; TLR4; GBP1; GBP4; CCR2; TLR2
Regulation of inflammatory response (GO:0050727)	20 of 249	0.0000025	CLEC12A; IL15; TRADD; NLRC3; FPR2; GATA3; NLRC4; USP18; HCK; AIM2; CASP5; MDK; CASP1; S100A12; FFAR2; TLR7; JAK2; TLR4; CCR2; TLR2
Regulation of TNF production (GO:0032680)	14 of 131	0.0000031	ARG2; NLRC3; LILRB4; LILRA4; TLR1; OAS1; OAS3; TNFRSF8; JAK2; FCGR1A; TLR4; TMEM106A; CCR2; TLR2
Positive regulation of inflammatory response (GO:0050729)	13 of 115	0.0000038	IL15; TRADD; NLRC4; AIM2; CASP5; MDK; CASP1; S100A12; TLR7; TLR4; GBP1; CCR2; TLR2
Regulation of ILβ production (GO:0032651)	11 of 82	0.0000039	AIM2; CASP1; TLR8; CD36; CARD16; TLR6; NLRC4; JAK2; TLR4; LILRB4; TMEM106A

GO biological process terms significantly enriched among the analyzed gene set are shown.

Consistent with these findings, the supplementation-associated candidate genes clustered into coherent immune-regulatory categories, including innate immune sensing (*TLR8*, *TLR10*, *CLEC12A*), immune inhibitory signaling (*SIGLEC6*, *CD300E*), interferon-associated pathways (*USP18*, *AZI2*), and immune-cell differentiation (*TBX21* (T-Box transcription factor 21)), while *SAT1* and *ZBTB16* represented established vitamin D target genes (**Figure 3C**). More broadly, GO analyses across candidate supplementation-responsive genes, RNA-seq/ATAC-seq overlap genes, genes associated with differentially accessible chromatin regions, and prioritized VDR-associated candidates consistently converged on innate immune sensing, pattern-recognition receptor signaling, cytokine communication, inflammatory regulation, leukocyte activation, and immune-cell differentiation processes (**Supplementary Table 4**). Collectively, these findings suggest that vitamin D_3_ supplementation was associated with preferential modulation of coordinated immune-regulatory programs rather than inducing broad transcriptome-wide remodeling.

In summary, the transcriptomic and enrichment analyses suggest that supplementation-associated candidate responses in the high-dose MS group were enriched for genes involved in innate immune sensing, interferon-associated signaling, and immune-regulatory pathways. Rather than producing global transcriptional remodeling, the observed candidate-gene patterns are consistent with effects on specific immune-related pathways relevant to MS-associated immune dysfunction.

### Epigenome-wide profiling reveals cohort-specific differences in PBMC chromatin accessibility

Aliquots of the same PBMC samples used for transcriptome analysis were subjected to epigenome-wide profiling of chromatin accessibility using ATAC-seq. Due to quality-control constraints, only paired baseline and follow-up samples from six MS patients taking daily high-dose vitamin D_3_, four MS patients receiving regular-dose vitamin D_3_ supplementation, and 29 healthy individuals receiving a monthly vitamin D_3_ bolus met the required criteria. Although the number of MS samples available for ATAC-seq was limited by stringent quality-control criteria, the paired longitudinal design allowed each participant to serve as their own baseline control, partially mitigating the loss of statistical power for detecting supplementation-associated chromatin accessibility changes. The integrated analysis of these 78 ATAC-seq datasets identified 18,998 consensus regions of accessible chromatin (CPM > 10) across PBMCs from all study participants (**Supplementary Table 3**). Of these, 10,794 regions were classified as parts of a transcription start site (TSS) region (± 500 bp from the TSS), while the remaining 8,204 regions were classified as distal accessible regions.

In the vicinity of the 18,998 accessible chromatin regions, 12,730 distinct genes were identified, of which 9,984 are protein-coding. Among these, 9,010 genes are expressed in PBMCs (**Supplementary Table 2**). Of the PBMC-expressed protein-coding genes, 5,832 harbor accessible chromatin exclusively at their TSS, 2,175 at both TSS and distal accessible regions, and 1,003 exclusively at distal accessible regions. In contrast, 4,031 PBMC-specifically expressed protein-coding genes are not located in proximity to any prominent region of chromatin accessibility.

**Figure 4:**
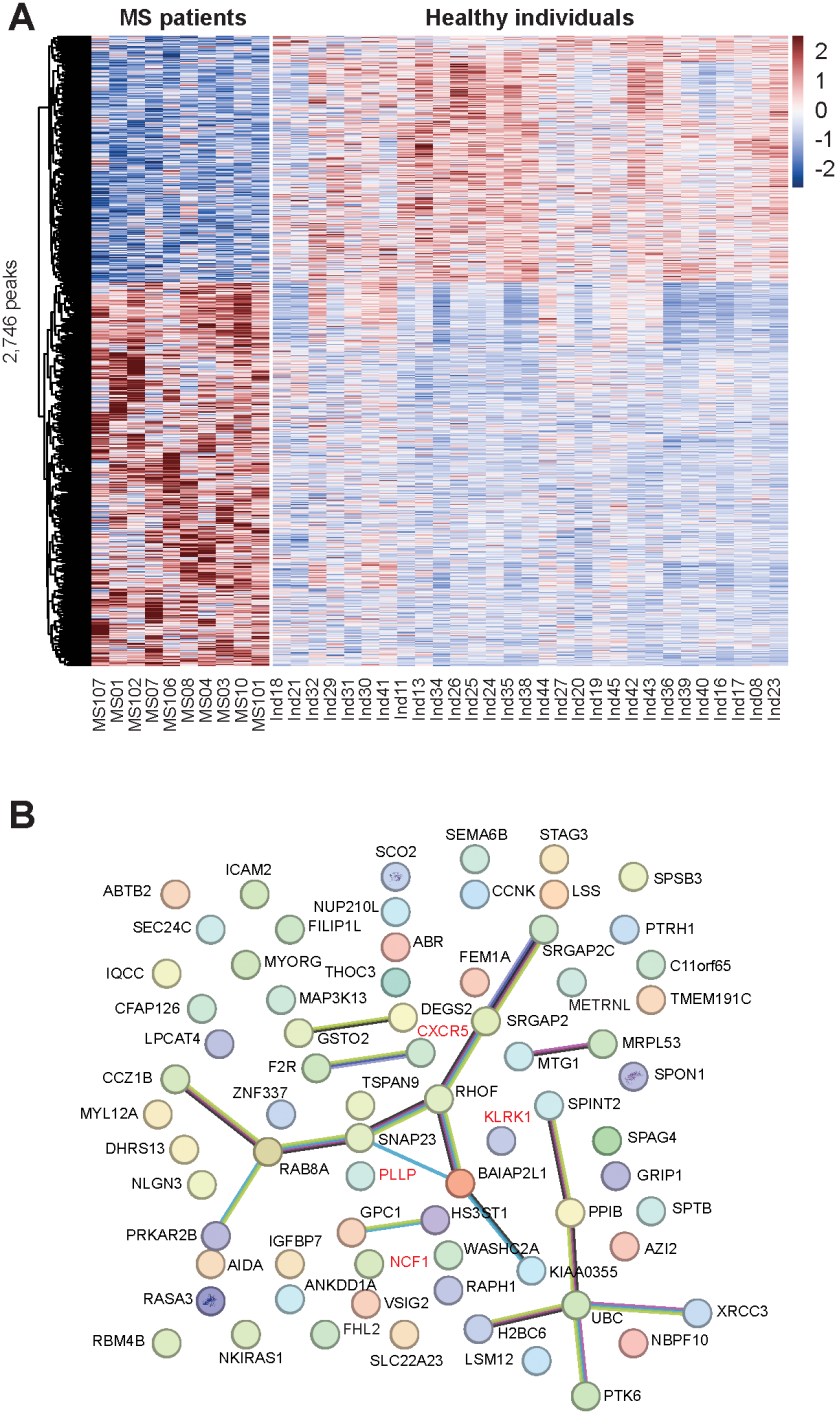
Chromatin accessibility differences between MS patients and healthy individuals. Heatmap of 2,746 differentially accessible chromatin regions identified by ATAC-seq analysis between MS patients (n = 10) and healthy individuals (n = 29) at baseline **(A)**. Differential accessibility was assessed using DiffBind with DESeq2-based statistical testing, and peaks were selected at FDR < 0.05. Heatmap values represent normalized chromatin accessibility signals. Rows and columns were hierarchically clustered using Euclidean distance and complete linkage. STRING protein-protein interaction network of genes associated with differentially accessible chromatin regions **(B)**. Selected immune-related genes discussed in the manuscript are highlighted in red. Network visualization is shown as a descriptive representation of known and predicted protein-protein interactions.

Hierarchical clustering of baseline chromatin accessibility profiles from all 39 study participants, combined with *k-means* clustering, clearly separated the 10 MS patients from the 29 healthy individuals (**Supplementary Figure 8**). Applying a statistical threshold of p_adj_ < 0.05, we identified 2,746 regions with differential chromatin accessibility between MS patients and healthy individuals (**Figure 4A**). Differential accessibility analyses were interpreted primarily at the level of convergent biological patterns and overlap with transcriptomic and external regulatory datasets rather than as evidence for isolated locus-specific regulatory effects. In total, 1,655 regions overlap with a TSS and 1,091 correspond to distal accessible regions. These differentially accessible regions are located in the vicinity of 2,459 genes, including 1,823 protein-coding genes expressed in PBMCs. Notably, 73 of these genes overlap with MS-specific genes identified by transcriptome analysis of the same groups (**Figure 2**).

To compare the overall strength of chromatin accessibility signals across all ATAC-seq contrasts, volcano plots were generated for each differential accessibility analysis (**Supplementary Figure 9**). These comparisons suggested marked differences in statistical support between disease-associated and supplementation-associated chromatin accessibility changes. The longitudinal analyses within MS patients receiving either daily high-dose (**Supplementary Figure 9A**) or regular vitamin D_3_ supplementation (**Supplementary Figure 9B**) yielded only nominally significant changes and no regions that remained significant after multiple-testing correction. In contrast, the healthy cohort displayed substantially larger supplementation-associated chromatin accessibility responses (**Supplementary Figure 9C**). Furthermore, the comparison between MS patients and healthy individuals identified 2,746 regions with FDR-significant differential accessibility (**Supplementary Figure 9D**). These observations indicate that chromatin accessibility differences associated with MS status are considerably more robust than the longitudinal chromatin accessibility changes observed following vitamin D_3_ supplementation in MS patients.

The STRING database was used to assess potential functional relationships among the proteins encoded by the 73 genes shared between chromatin accessibility and transcriptome analyses. Interactions were identified for only 20 of these proteins, while the majority remained disconnected under the applied STRING confidence settings (**Figure 4B**). Thus, the overlapping chromatin accessibility and transcriptomic signatures do not converge on a single coherent interaction network but instead suggest heterogeneous and partially independent regulatory processes. Motif enrichment analysis of the vitamin D-associated accessibility changes in the high-dose MS group identified several nominally enriched motifs; however, none remained significant after Benjamini-Hochberg correction for multiple testing. Given the limited number of differentially accessible regions and the small number of paired ATAC-seq samples available for analysis, these findings were not interpreted further.

Several of the identified genes have previously been implicated in MS pathophysiology. These include *NCF1* (neutrophil cytosolic factor 1), a key component of the NADPH oxidase complex involved in myeloid cell-derived reactive oxygen species production, *CXCR5* (C-X-C motif chemokine receptor 5), a chemokine receptor central to B cell and T follicular helper cell trafficking, *KLRK1* (killer cell lectin like receptor K1), encoding the activating NK and CD8^+^ T-cell receptor NKG2D, and *PLLP* (plasmolipin), a myelin-associated gene enriched in oligodendrocytes. The presence of MS-associated chromatin accessibility changes in regulatory regions proximal to these genes is consistent with potential involvement in disease-specific immune and neurobiological alterations observed in this group.

Taken together, epigenome-wide chromatin accessibility profiling suggested a strong and stable regulatory distinction between the MS and healthy cohorts characterized by thousands of differentially accessible regions and partial concordance with disease-associated gene expression changes. By comparison, vitamin D_3_ supplementation induced only limited chromatin accessibility changes in MS patients, suggesting that supplementation-associated epigenetic effects are subtle and exploratory relative to the robust disease-associated chromatin landscape.

### Integrative analysis prioritizes candidate vitamin D-responsive genes with MS-relevant functions

**Figure 5:**
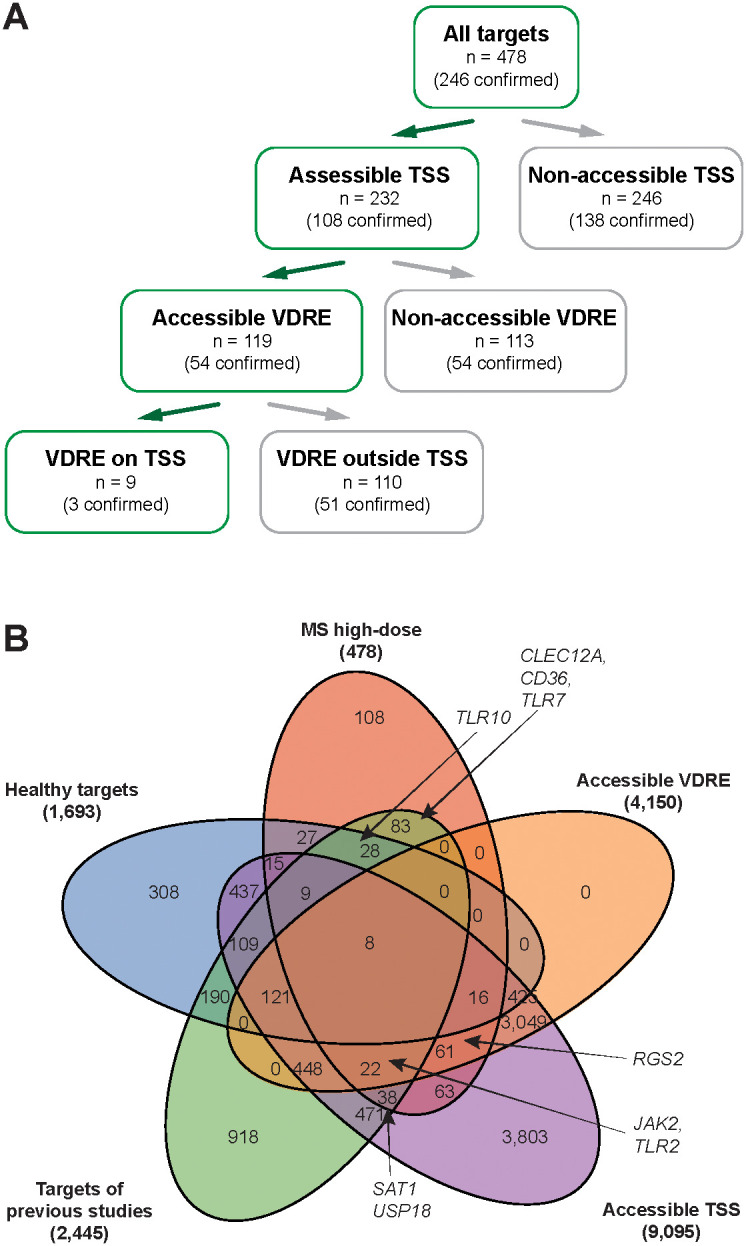
Integration of transcriptomic, epigenomic, and VDR-binding datasets identifies candidate vitamin D target genes in MS. Workflow for prioritization of supplementation-associated candidate genes **(A)**. Genes were initially filtered using nominal p < 0.05, |log_2_FC| > 0.25, and log_2_CPM > 1. Accessible chromatin regions were assigned based on overlap with the TSS (± 500 bp) or distal regulatory regions within ±500 kb of the corresponding gene locus. Published VDR ChIP-seq datasets were used as an additional prioritization criterion. Numbers in parentheses indicate genes confirmed by overlapping transcriptomic, chromatin accessibility, and VDR-binding evidence. Selected prioritized candidate genes grouped according to accessibility status and VDR association **(B)**. The integration strategy was used as a descriptive framework for prioritizing biologically plausible vitamin D-responsive candidate genes and was not treated as a formal statistical enrichment analysis.

To prioritize supplementation-associated candidate genes with the strongest support for direct regulatory involvement, we integrated transcriptome, chromatin accessibility, published VDR ChIP-seq (chromatin immunoprecipitation sequencing), and previously validated vitamin D target datasets in a stepwise filtering framework (**Figure 5**). Among the 478 candidate supplementation-responsive genes identified in PBMCs from MS patients taking daily high-dose vitamin D_3_ supplementation, 232 genes were associated with prominently accessible chromatin at their TSS, of which 108 had already been independently reported as vitamin D-responsive in previous studies (**Figure 5A**). In parallel, integration with published VDR ChIP-seq datasets from THP-1 cells (36, 37) identified 119 genes with VDR occupancy within accessible chromatin regions located within ±500 kb of the TSS, including 54 genes supported by independent validation datasets. This proximity-based assignment was used solely for candidate-gene prioritization and should not be interpreted as evidence of direct regulatory interactions between VDR-binding regions and the associated genes.

Further stratification of the VDR-associated genes demonstrated that only nine candidates displayed VDR occupancy directly at their annotated TSS, whereas the remaining 110 genes contained VDR-bound regions at more distal regulatory sites (**Figure 5B**). Importantly, these candidate genes were prioritized through convergence of independent evidence layers, including longitudinal transcriptional responsiveness, chromatin accessibility, proximity to accessible regulatory regions, previously reported VDR occupancy, and reproducibility across external vitamin D datasets. Of these genes, 54 genes were supported by independent experimental datasets. Notably, comparison with previously published vitamin D target datasets demonstrated that more than half of the high-dose-responsive genes lacked prior experimental support, emphasizing the added value of longitudinal *in vivo* profiling for identifying context-dependent supplementation-associated candidate genes in MS PBMCs.

Because the VDR occupancy information was derived from THP-1 monocytes rather than matched PBMC samples, these overlaps should be interpreted as supportive prioritization signals rather than definitive evidence of direct VDR-mediated regulation *in vivo*. A subset of nine genes fulfilled the most stringent prioritization criteria by combining vitamin D responsiveness, accessible promoter chromatin, direct VDR occupancy at the TSS, and partial validation in independent datasets, thereby representing the highest-priority candidate genes for future validation studies. These genes include *ABCA2* (ATP binding cassette subfamily A member 2), *AGFG2* (ArfGAP with FG repeats 2), *AKR1B1* (aldo-keto reductase family 1 member B), *AZI2*, *BCL9L* (BCL9 like), *BICDL1* (BICD family like cargo adaptor 1), *DUSP6* (dual specificity phosphatase 6), *MFSD3* (major facilitator superfamily domain-containing protein 3), and *SIGLEC6*. Notably, several of these prioritized candidates (*ABCA2*, *AZI2*, and *SIGLEC6*) were among the genes highlighted in the differential-expression volcano plots (**Figure 3A**), providing independent support from both transcriptional and regulatory prioritization analyses. Furthermore, the genes *BICDL1*, *DUSP6*, and *SIGLEC6* had already been validated as VDR targets in independent systems. Among these nine candidates, *AZI2*, *ABCA2*, and *SIGLEC6* are linked to MS-relevant biological pathways. *AZI2* encodes an adaptor protein involved in innate immune signaling and regulates type I interferon and NFκB activation, pathways known to be dysregulated in MS. *ABCA2*, a lipid transporter highly expressed in the central nervous system, has been reported to show altered expression in MS brain tissue and has been implicated in myelin-associated processes (42). *SIGLEC6* encodes an inhibitory immune receptor expressed on subsets of B cells and myeloid cells and belongs to a receptor family involved in immune regulation and tolerance mechanisms relevant to MS pathophysiology (43). Together, these observations support prioritization of *AZI2*, *ABCA2*, and *SIGLEC6* as biologically plausible candidate genes for future mechanistic investigation but do not establish direct VDR-mediated regulation in PBMCs.

In summary, integrative analysis of chromatin accessibility and published VDR ChIP-seq data refined the 478 supplementation-associated candidate genes to a prioritized subset with accessible promoters and VDR occupancy. This analysis prioritized nine candidate genes, including *AZI2*, *ABCA2*, and *SIGLEC6*, that converge on innate immune, interferon, and myelin-associated pathways relevant to MS pathophysiology.

## Discussion

In this study, we integrated longitudinal transcriptomic and epigenomic profiling of PBMCs to investigate how distinct vitamin D_3_ supplementation regimens are associated with immune-related molecular responses in MS. MS patients exhibited stable transcriptional and chromatin-accessibility profiles that remained distinct from those observed in the healthy cohort throughout the study period. Rather than inducing a global transcriptomic shift, vitamin D_3_ supplementation was associated with selective changes in immune-related gene expression that varied across the analyzed study groups. These findings are consistent with recent reviews highlighting immune dysregulation, disease heterogeneity, and the need for biomarker-guided precision approaches in MS (44), as well as with clinical studies showing that high-dose vitamin D_3_ supplementation can induce molecular immune responses without immediate clinical benefit (45, 46). Because disease-modifying therapies were not experimentally controlled and participant numbers were limited, potential interactions between vitamin D_3_ supplementation and specific treatment regimens could not be evaluated. Future studies with larger cohorts will be required to determine how individual disease-modifying therapies influence supplementation-associated molecular responses.

Collectively, our exploratory analyses suggest that vitamin D_3_ supplementation may be associated with selective modulation of interconnected immune-regulatory pathways rather than generalized transcriptional reprogramming of PBMCs. Importantly, because no supplementation-associated transcriptomic changes remained significant after correction for multiple testing, the biological interpretations presented here are based on exploratory candidate-gene analyses and should be regarded as hypothesis-generating rather than confirmatory evidence of vitamin D-dependent molecular regulation. The most consistently affected genes and enriched biological processes were related to innate immune sensing, pattern-recognition receptor signaling, cytokine-mediated communication, interferon-associated responses, and immune-cell differentiation. Notably, genes such as *TLR8*, *TLR10*, *CLEC12A*, *USP18*, *AZI2*, *SAT1*, and *ZBTB16* point toward coordinated regulation of pathways involved in immune activation and immune homeostasis. These observations are consistent with the established role of vitamin D as a context-dependent modulator of immune function rather than a broad immunosuppressive signal.

Importantly, the observational design of this study limits causal interpretation of these findings. The high-dose supplementation group was self-selected, supplementation doses varied among participants, and the healthy comparison cohort differed from the MS cohort with respect to disease status, age, baseline vitamin D status, supplementation regimen, and follow-up protocol. Consequently, the observed transcriptional and chromatin accessibility differences cannot be attributed exclusively to vitamin D_3_ supplementation and may also reflect underlying biological, clinical, or environmental differences between cohorts. Therefore, cross-cohort comparisons were used primarily to identify candidate regulatory patterns for future investigation rather than to establish direct effects of vitamin D_3_. Moreover, the high-dose supplementation group encompassed a broad range of daily vitamin D_3_ doses (20,000-70,000 IU/day), introducing additional heterogeneity that may have contributed to inter-individual variability and reduced the ability to detect consistent supplementation-associated molecular responses.

Despite substantial increases in serum 25(OH)D_3_ concentrations, which varied considerably between participants, and reduced PTH levels in patients receiving daily high-dose supplementation, the transcriptional and chromatin-accessibility differences observed between the cohorts remained largely unchanged over the three-month observation period. MS patients continued to display broadly similar patterns of elevated expression of immune-related genes, particularly within the *HLA* locus and additional immune-associated gene families, indicating that short-term vitamin D_3_ supplementation was not associated with major alterations of the underlying disease-associated immune state in peripheral blood. This persistence is consistent with the absence of detectable changes in disability, neuroaxonal injury, or functional performance during the study period and supports the view that molecular immune responses may precede measurable clinical effects (47).

Importantly, the supplementation-associated candidate gene sets identified in MS patients and healthy individuals showed limited overlap. Only 103 of the 478 exploratory supplementation-associated candidate genes identified in the daily high-dose MS group overlapped with those detected in healthy participants, despite comparable analytical workflows. Nevertheless, several previously validated *in vivo* vitamin D target genes, including *TBX21*, *ZBTB16*, and *SAT1*, were identified among the responsive genes, indicating that canonical vitamin D signaling remains functional in MS. Functional enrichment analyses consistently highlighted innate immune sensing, Toll-like receptor signaling, cytokine-associated communication, inflammatory regulation, and leukocyte activation, suggesting that vitamin D responsiveness is preserved but shaped by the underlying disease-associated immune environment. Together, these are consistent with the possibility that vitamin D signaling in MS occurs within a pre-existing disease-associated regulatory environment (48, 49).

Seasonal variation in immune-cell function and vitamin D-responsive transcriptional programs may have contributed to inter-individual variability in the observed responses (50–52). Although the present study was not designed to formally assess seasonal effects, seasonality represents an additional source of biological heterogeneity that should be considered in future studies.

The overlap between transcriptomic and chromatin accessibility signatures was limited, with only 73 genes supported by both datasets. Although modest, this degree of concordance is consistent with previous multi-omic studies demonstrating that chromatin accessibility and transcription capture distinct but complementary layers of gene regulation (53). Chromatin accessibility changes may precede transcriptional responses, persist after transcriptional adaptation, or occur within specific immune-cell subsets that are not resolved in bulk PBMC analyses (54–56). Thus, limited overlap should not be interpreted as a lack of biological relevance but rather as a reflection of the complex and context-dependent nature of immune-cell regulation.

ATAC-seq nevertheless suggested extensive chromatin-accessibility differences between the MS and healthy cohorts involving genes linked to oxidative burst regulation, immune-cell trafficking, cytotoxic lymphocyte activation, and myelin biology (57, 58). Together with the sparse STRING interaction networks observed among overlapping transcriptomic and chromatin-associated genes, these findings support a model in which vitamin D_3_ modulates selected regulatory nodes within a pre-existing disease-associated chromatin landscape rather than inducing widespread epigenetic reprogramming. The limited chromatin accessibility response may further reflect the fact that vitamin D signaling can influence transcription through mechanisms requiring only subtle accessibility changes or through modulation of already accessible regulatory regions (59).

Among the candidate supplementation-responsive genes, innate immune and inflammatory pathways were consistently represented. Recurrent identification of TLR family members together with *TBX21*, *ZBTB16*, *SAT1*, *AZI2*, and *SIGLEC6* supports the hypothesis that vitamin D_3_ exposure may be associated with selected immune-regulatory programs in PBMCs. However, because these findings are derived from exploratory candidate-gene analyses, they require validation in independent cohorts (60).

Integration of transcriptomic and epigenomic data with published VDR occupancy datasets (36, 37) further prioritized a limited subset of candidate genes potentially associated with vitamin D signaling supported by multiple lines of evidence. Among these, *AZI2*, *ABCA2*, and *SIGLEC6* are of particular interest because they connect vitamin D responsiveness to innate immune signaling, myelin-associated biology, and inhibitory immune receptor pathways relevant to MS (61). However, because VDR occupancy was inferred from published THP-1 datasets rather than matched PBMC VDR profiling, these assignments should be regarded as supportive prioritization signals rather than evidence of direct regulatory relationships and therefore require experimental validation in future cell-type-specific studies.

Several limitations should be considered when interpreting these findings. First, the small sample size, particularly the six paired ATAC-seq datasets available after quality-control filtering, substantially limits statistical power and increases the likelihood of both false-negative and false-positive findings. Consequently, individual candidate genes and chromatin regions should be regarded as provisional observations requiring validation in larger cohorts. Second, the study was observational rather than randomized. Participants self-selected their supplementation regimens, and the high-dose group encompassed a broad range of daily vitamin D_3_ doses (20,000-70,000 IU/day), introducing additional exposure heterogeneity that may have contributed to inter-individual variability and reduced the ability to detect consistent supplementation-associated molecular responses. Consequently, the observed molecular signatures cannot be attributed exclusively to vitamin D_3_ supplementation and may also reflect other biological, clinical, or environmental factors. Third, comparisons between MS patients and healthy individuals should be interpreted with caution. The healthy cohort originated from an independent intervention study and differed from the MS cohort with respect to disease status, age, baseline vitamin D status, supplementation protocol, and PBMC processing procedures. In addition, follow-up intervals differed between cohorts, averaging 129 ± 29 days in MS patients and 84 days in healthy individuals. Therefore, differences observed between cohorts may reflect not only biological context and supplementation regimen but also differences in study design and exposure duration. Furthermore, because the cohorts were recruited in different countries and study settings, unmeasured cohort-specific effects cannot be excluded despite the use of comparable sequencing platforms and analytical workflows. Fourth, the VDR-associated prioritization framework relied on published VDR ChIP-seq datasets generated in THP-1 cells rather than matched PBMC samples from study participants. Because VDR binding patterns are known to be cell-type dependent, the identified overlaps should be regarded as indirect supportive evidence for candidate prioritization rather than as evidence of direct VDR occupancy or regulation in PBMCs. Finally, PBMCs represent a heterogeneous mixture of immune-cell populations, limiting resolution of cell-type-specific transcriptional and chromatin accessibility responses. Future studies employing larger cohorts, randomized controlled supplementation designs, and cell-type-resolved multi-omics approaches will be required to validate the candidate genes and regulatory pathways identified here and to establish their mechanistic relevance for vitamin D-mediated immune regulation in MS.

In conclusion, our integrative longitudinal multi-omics analysis indicates that MS is characterized by stable disease-associated transcriptional and chromatin accessibility signatures in peripheral immune cells, while vitamin D_3_ supplementation is associated with selective, context-dependent molecular responses rather than global immune reprogramming. Although no longitudinal transcriptomic changes remained significant after correction for multiple testing, daily high-dose vitamin D_3_ supplementation was associated with exploratory candidate molecular responses involving innate immune and inflammatory pathways and identified a subset of candidate VDR-associated regulatory targets relevant to MS immunopathology, although without detectable short-term clinical effects. These findings provide a framework for prioritizing candidate vitamin D-responsive pathways and genes for mechanistic studies but require validation in larger prospective cohorts and randomized controlled intervention studies.

## Data Availability

The datasets presented in this study can be found in online repositories. The names of the repository/repositories and accession number(s) can be found in the article/**Supplementary Material**.

## Glossary

1,25(OH)_2_D_3_: 1α,25-dihydroxyvitamin D_3_
25(OH)D_3_: 25-hydroxyvitamin D_3_
9HPT: 9-hole peg test
ABCA2: ATP binding cassette subfamily A member 2
AGFG2: ArfGAP with FG repeats 2
AKR1B1: aldo-keto reductase family 1 member B
ARR: annualized relapse rate
ATAC-seq: assay for transposase accessible chromatin with high-throughput sequencing
AZI2: 5-azacytidine induced 2
BCL9L: BCL9 like
BICDL1: BICD family like cargo adaptor 1
BMI: body mass index
CD300: CD300 molecule like family member
CD36: CD molecule
ChIP-seq: chromatin immunoprecipitation sequencing
CLEC12A: C-type lectin domain family 12 member A
CPM: counts per million
CR1: complement C3b/C4b receptor 1
CXCR5: C-X-C motif chemokine receptor 5
DUSP6: dual specificity phosphatase 6
EDSS: expanded disability status scale
FC: fold change
FDR: false discovery rate
GO: gene ontology
GOLGA8: golgin A8 family member
HGNC: Human Gene Nomenclature Committee
HLA: major histocompatibility complex, also called human leukocyte antigen
IQR: interquartile range
JAK2: janus kinase 2
KLRK1: killer cell lectin like receptor K1
LILR: leukocyte immunoglobulin like receptor
MDS: multidimensionality scaling
MFSD3: major facilitator superfamily domain-containing protein 3
MRI: magnetic resonance imaging
MS: multiple sclerosis
NBPF: NBPF member
NCF1: neutrophil cytosolic factor 1
NK: natural killer
NLRC4: NLR family CARD domain containing 4
NPIP: nuclear pore complex interacting protein family member
PBMC: peripheral blood mononuclear cell
PBS: phosphate-buffered saline
PLLP: plasmolipin
PTH: parathyroid hormone
PVRIG: PVR related immunoglobulin domain containing
RNA-seq: RNA sequencing
RRMS: relapsing-remitting multiple sclerosis
SAT1: spermidine/spermine N1-acetyltransferase 1
SDMT: symbol digit modalities test
SIGLEC6: sialic acid binding Ig like lectin 6
sNfL: serum neurofilament light chain
SPDYE: speedy/RINGO cell cycle regulator family member
SURF: surfeit
T25FW: timed 25-foot walk
TBC1D3: TBC1 domain family member
TBX21: T-Box transcription factor 21
TLR: Toll-like receptor
TNF: tumor necrosis factor
TSS: transcription start site
USP18: ubiquitin specific peptidase 18
VDR: vitamin D receptor
ZAP70: zeta chain of T cell receptor associated protein kinase 70
ZBTB16: zinc finger and BTB domain containing 16
