# Supplementary material for: Exploratory multi-omics analysis suggests context-dependent immune responses to vitamin D3 supplementation in PBMCs of multiple sclerosis patients
Source: Front Immunol. 2026 Jul 31;17:1845005. doi: 10.3389/fimmu.2026.1845005 (PMC13472867; doi:10.3389/fimmu.2026.1845005)
Supplement: Supplementary file 1 [file DataSheet1.pdf]

SUPPLEMENTARY DATA

SUPPLEMENTARY TABLES

Table S1. Read alignment statistics. The number of uniquely aligned reads per sample is provided for both ATAC-seq and RNA-seq datasets.

Table S2. Transcriptomic changes in PBMCs. PBMCs were isolated at day 0, and day 84 of 17 MS patients and 42 healthy individuals. RNA-seq analysis identified 13,041 expressed protein-coding genes (CPM > 1).

Table S3. Epigenomic changes in PBMCs. PBMCs were isolated at day 0, and day 84 of 10 MS patients and 29 healthy individuals. ATAC-seq analysis identified 18,998 prominent regions of accessible chromatin (CPM > 10).

Table S4. GO enrichment analysis of transcriptomic and epigenomic gene sets. GO Biological Process enrichment analyses were performed for MS-associated differentially expressed genes, vitamin D_3-responsive genes identified in each supplementation group, genes overlapping between RNA-seq and ATAC-seq analyses, genes associated with differentially accessible chromatin regions, and prioritized VDR-associated candidate target genes. For each gene set, significantly enriched GO terms are listed together with the corresponding GO identifier, gene count, enrichment statistics, and adjusted p-values.

SUPPLEMENTARY FIGURES

Figure S1. Clustering of MS-associated transcriptomic signatures at baseline and after vitamin D_3 supplementation. Heatmaps of the 1,480 genes differentially expressed between MS patients and healthy individuals (FDR < 0.05) at baseline (**A**, day 0) and after 84 days of supplementation (**B**, day 84). RNA-seq data were obtained from 17 MS patients and 42 healthy individuals. Expression values represent CPM-normalized counts subjected to per-gene min-max normalization (0-1 scaling). Genes were grouped into ten clusters based on hierarchical clustering using Euclidean distance and complete linkage. Samples are shown in the same order as in **Figure 2A**.

Figure S2. Expression of *HLA* genes in MS patients and healthy individuals. CPM-normalized expression levels of class I (*HLA-A, HLA-B, HLA-C, HLA-E, HLA-F, HLA-G*) and class II (*HLA-DMA, HLA-DMB, HLA-DOA, HLA-DOB, HLA-DPA1, HLA-DPB1, HLA-DQA1, HLA-DQB1, HLA-DQB2, HLA-DRA, HLA-DRB1, HLA-DRB5*) *HLA* genes in MS patients (n = 17) and healthy individuals (n = 42) at baseline. Boxes indicate median and interquartile range; dots represent individual samples.

Figure S3. Volcano plots of vitamin D-responsive genes identified by RNA-seq. Volcano plots showing differential gene expression between day 0 and day 84 in MS patients receiving regular vitamin D_3 supplementation (**A**, n = 7), and healthy individuals receiving monthly vitamin D_3 bolus supplementation (**B**, n = 42). The x-axis shows $\log_2$FC and the y-axis shows $-\log_{10}$(p-value). Red and blue dots indicate significantly upregulated and downregulated genes, respectively, defined by nominal $p < 0.05$ and $|\log_2FC| > 0.25$; gray dots indicate non-significant genes. Numbers of regulated and non-significant genes are indicated in each panel.

Figure S4. Estimated immune-cell composition of PBMC samples. Estimated fractions of monocytes, T cells, B cells, and NK cells in PBMC samples from healthy individuals, MS patients receiving regular vitamin D_3 supplementation, and MS patients receiving daily high-dose vitamin D_3 supplementation at day 0 and day 84. Cell fractions were inferred from RNA-seq data using transcriptome-based

deconvolution. Boxes indicate median and interquartile range; dots represent individual samples. NS, not significant.

Figure S5. MDS scaling analysis of transcriptomic profiles before and after vitamin D_3 supplementation. MDS scaling plots based on RNA-seq expression profiles from MS patients receiving daily high-dose vitamin D_3 supplementation (**A**), MS patients receiving regular vitamin D_3 supplementation (**B**), and healthy individuals receiving monthly vitamin D_3 bolus supplementation (**C**). Analyses were performed using either all expressed genes (left panels) or vitamin D target genes (right panels). Blue circles indicate baseline samples (day 0) and red triangles indicate samples collected after 84 days of supplementation (day 84). Numbers identify individual participants.

Figure S6. Correlation between changes in serum 25(OH)D_3 concentrations and gene expression. Correlation analysis between changes in serum 25(OH)D_3 concentrations (Δ25(OH)D_3, day 84 minus day 0) and corresponding changes in gene expression (Δexpression, day 84 minus day 0) in MS patients receiving daily high-dose vitamin D_3 supplementation. Selected genes showing Pearson correlation coefficients $|R| > 0.30$ are displayed. Each point represents one individual participant.

Figure S7. STRING protein-protein interaction network of MS-associated vitamin D-responsive genes. The network comprises 45 genes identified by overlap between the 478 vitamin D-responsive genes detected in MS patients receiving daily high-dose vitamin D_3 supplementation and the 1,480 genes differentially expressed between MS patients and healthy individuals at baseline. Nodes represent proteins encoded by the overlapping genes, and edges indicate known or predicted protein-protein interactions retrieved from the STRING database. Selected immune-regulatory and MS-relevant genes discussed in the manuscript are highlighted in red (*CR1*, *HLA-DPB1*, and *ZAP70*). The network is presented as a descriptive visualization of functional relationships among MS-associated vitamin D-responsive genes and does not imply causal interactions.

Figure S8. Global chromatin accessibility profiles in MS patients and healthy individuals. Heatmap showing normalized ATAC-seq accessibility values for 18,998 chromatin regions with CPM > 10 in PBMC samples from MS patients (n = 10) and healthy individuals (n = 29) at baseline. Accessibility values were normalized using the DiffBind workflow. Peaks and samples were hierarchically clustered using Euclidean distance and complete linkage. Red and blue colors indicate increased and decreased chromatin accessibility, respectively.

Figure S9. Differential chromatin accessibility across ATAC-seq comparisons. Volcano plots showing differential chromatin accessibility ($\log_2$FC versus $-\log_{10}$(p-value)) for MS patients receiving daily high-dose vitamin D_3 supplementation (**A**, n = 6 paired samples), MS patients receiving regular vitamin D_3 supplementation (**B**, n = 4 paired samples), healthy individuals receiving monthly vitamin D_3 bolus supplementation (**C**, n = 29 paired samples), and the baseline comparison between MS patients (n = 10) and healthy individuals (n = 29) (**D**). Differential accessibility was assessed using DiffBind with DESeq2-based statistical testing. Red dots indicate regions with increased accessibility (opening), blue dots indicate regions with decreased accessibility (closing), and gray dots indicate non-significant regions. The number of FDR-significant regions (FDR < 0.05) is indicated in each panel. No FDR-significant accessibility changes were observed in the longitudinal MS analyses, whereas robust accessibility differences were detected between MS patients and healthy individuals and following monthly vitamin D_3 bolus supplementation in healthy individuals.

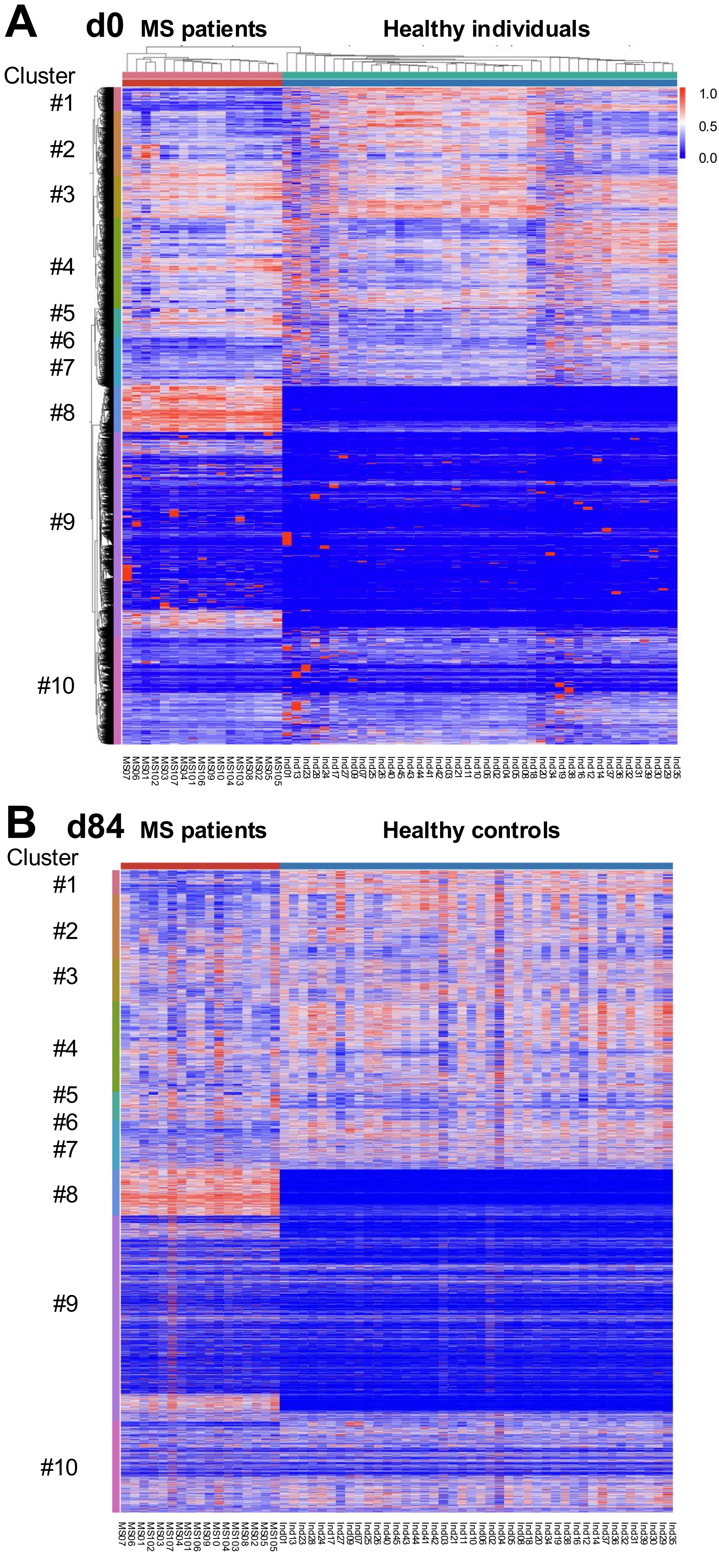

A
d0
MS patients
Healthy individuals
Cluster
#1
#2
#3
#4
#5
#6
#7
#8
#9
#10
1.0
0.5
0.0
B
d84
MS patients
Healthy controls
Cluster
#1
#2
#3
#4
#5
#6
#7
#8
#9
#10

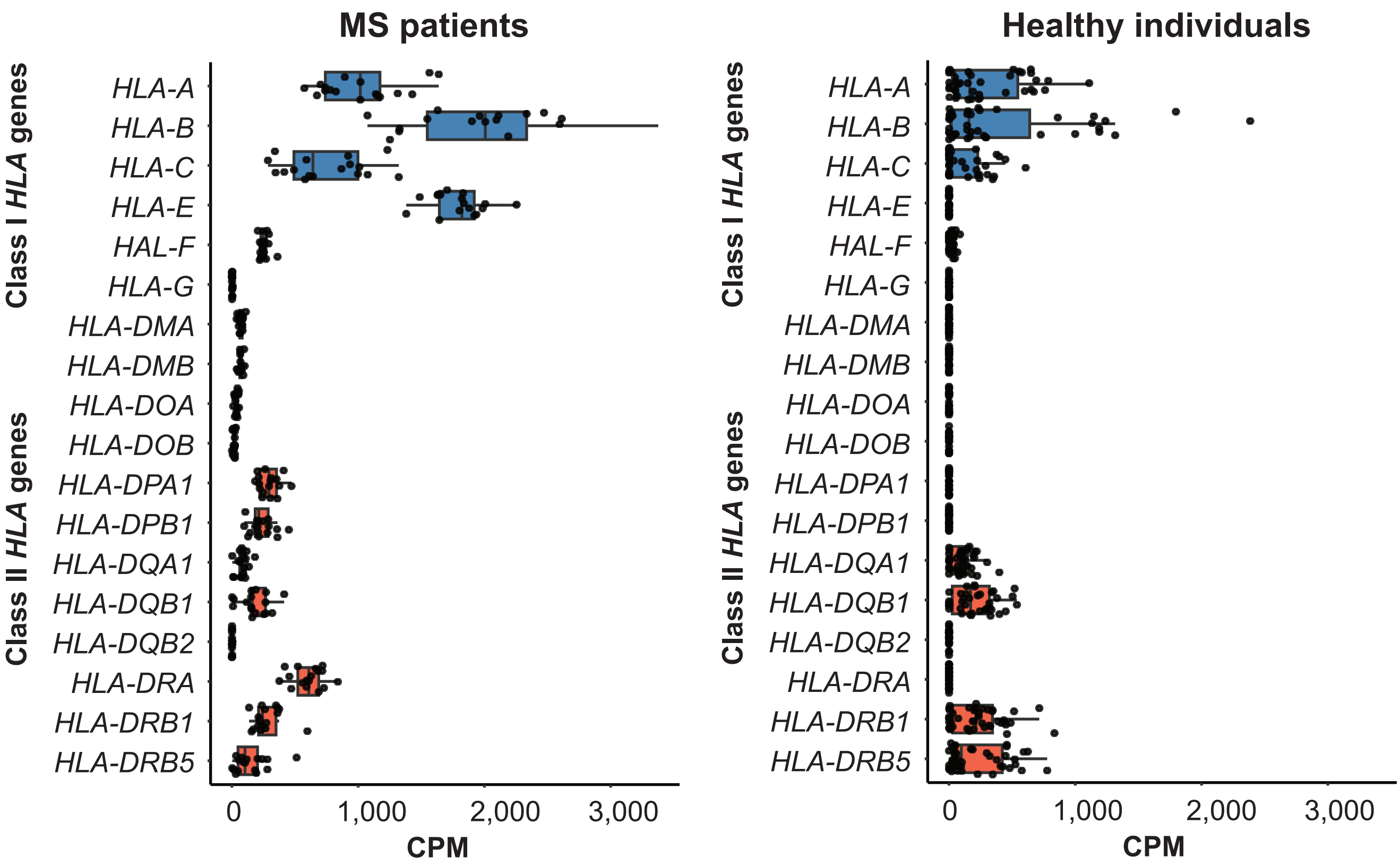
MS patients
Healthy individuals
Class I HLA genes
Class II HLA genes
HLA-A
HLA-B
HLA-C
HLA-E
HAL-F
HLA-G
HLA-DMA
HLA-DMB
HLA-DOA
HLA-DOB
HLA-DPA1
HLA-DPB1
HLA-DQA1
HLA-DQB1
HLA-DQB2
HLA-DRA
HLA-DRB1
HLA-DRB5
0
1,000
2,000
3,000
CPM

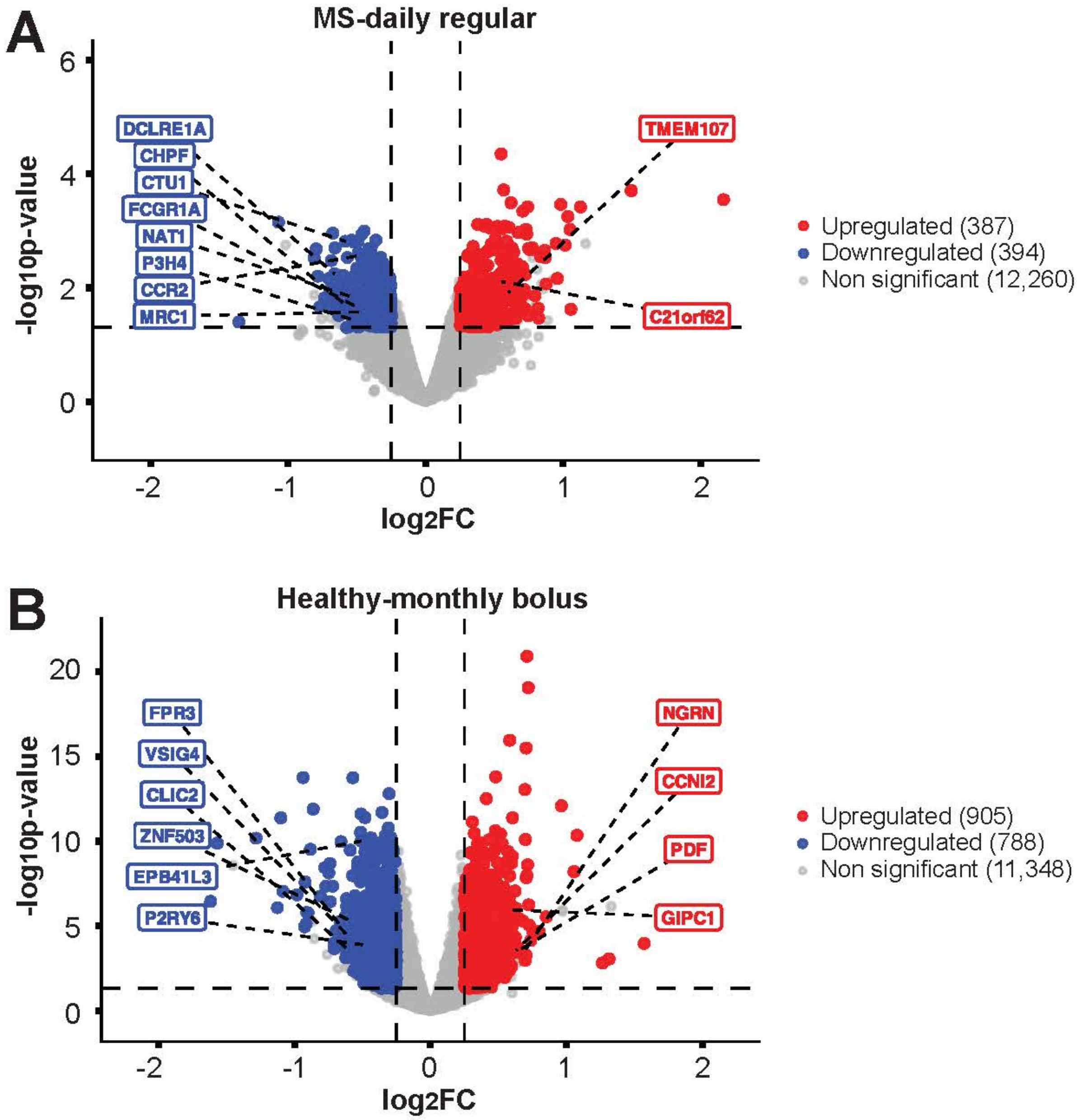
A
MS-daily regular
-log10p-value
log2FC
DCLRE1A
CHPF
CTU1
FCGR1A
NAT1
P3H4
CCR2
MRC1
TMEM107
C21orf62
Upregulated (387)
Downregulated (394)
Non significant (12,260)
B
Healthy-monthly bolus
-log10p-value
log2FC
FPR3
VSIG4
CLIC2
ZNF503
EPB41L3
P2RY6
NGRN
CCNI2
PDF
GIPC1
Upregulated (905)
Downregulated (788)
Non significant (11,348)

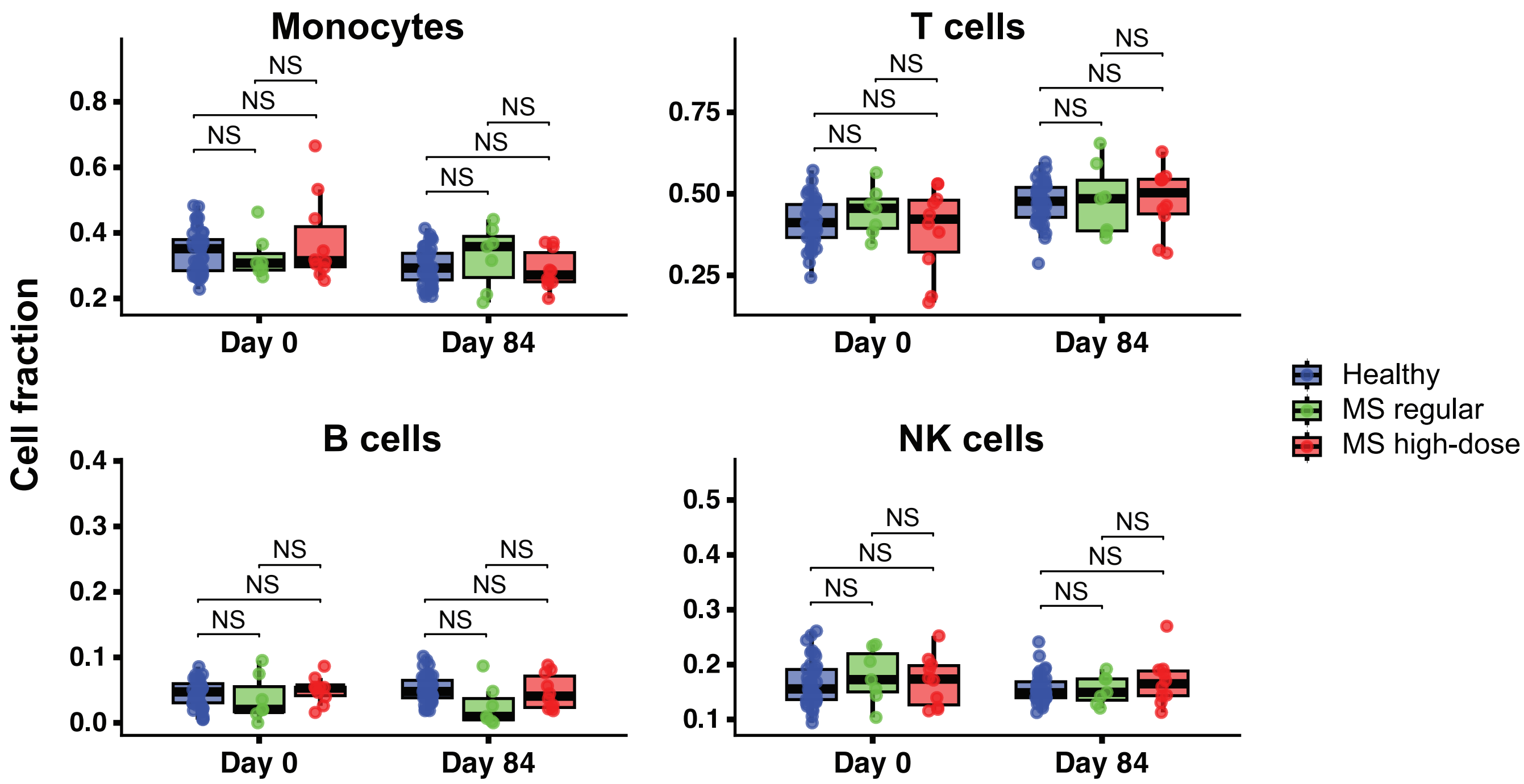
Monocytes
T cells
B cells
NK cells
Cell fraction
NS
Day 0
Day 84
0.8
0.6
0.4
0.2
0.75
0.50
0.25
0.3
0.1
0.0
0.5
Healthy
MS regular
MS high-dose

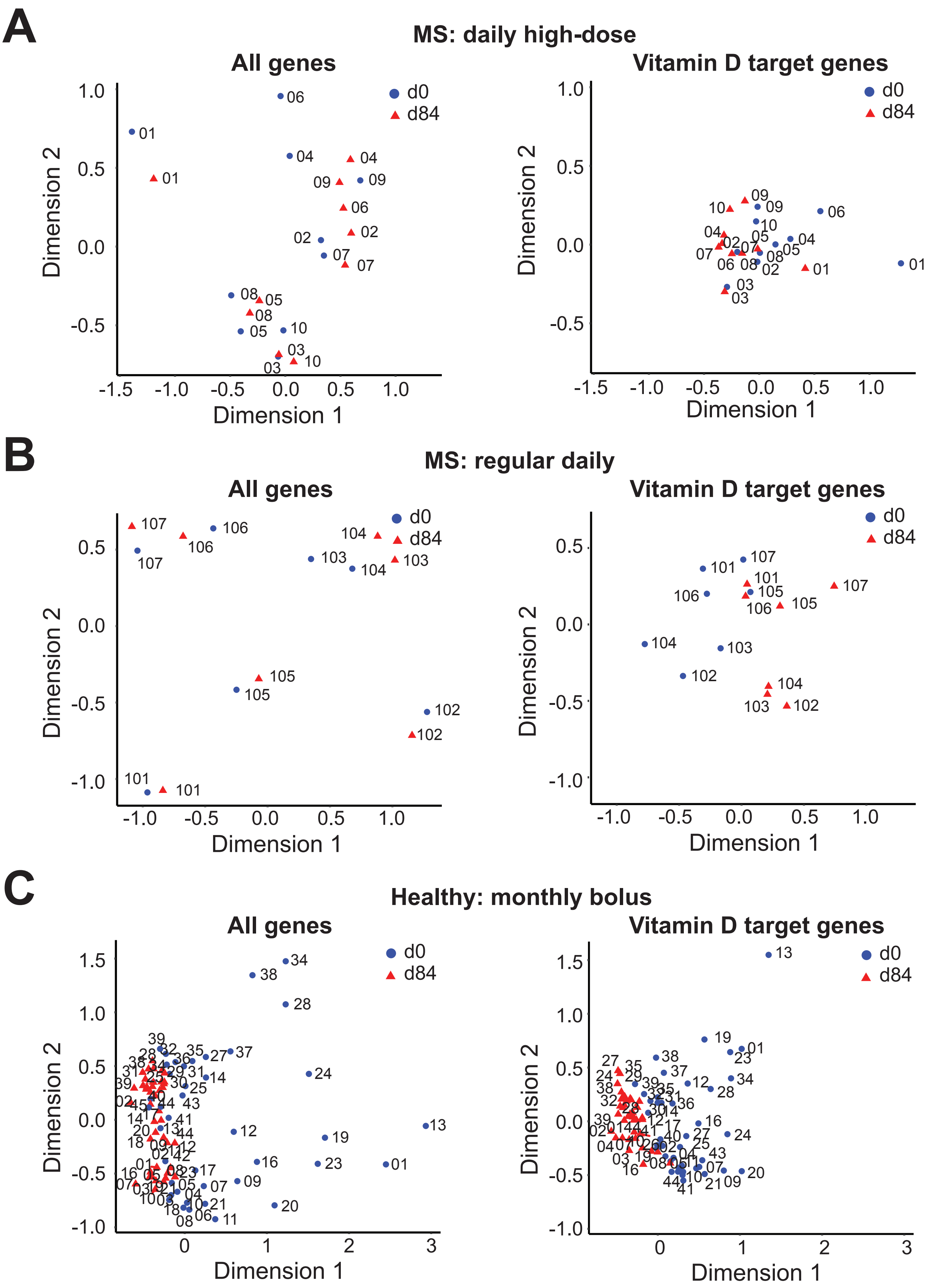
A
MS: daily high-dose
All genes
Vitamin D target genes
d0
d84
Dimension 1
Dimension 2
B
MS: regular daily
All genes
Vitamin D target genes
d0
d84
Dimension 1
Dimension 2
C
Healthy: monthly bolus
All genes
Vitamin D target genes
d0
d84
Dimension 1
Dimension 2

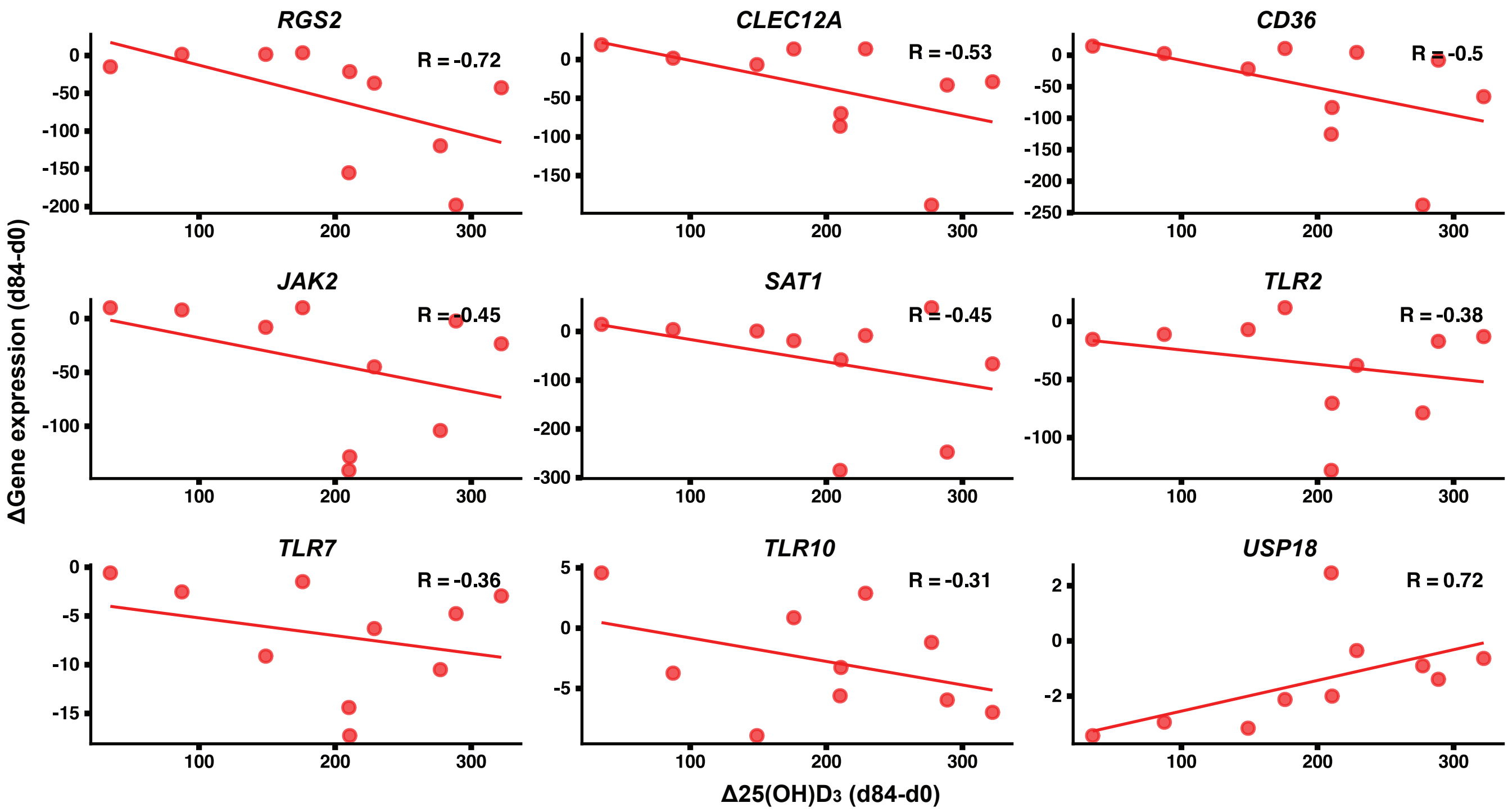
RGS2
R = -0.72
CLEC12A
R = -0.53
CD36
R = -0.5
JAK2
R = -0.45
SAT1
R = -0.45
TLR2
R = -0.38
TLR7
R = -0.36
TLR10
R = -0.31
USP18
R = 0.72
ΔGene expression (d84-d0)
Δ25(OH)D3 (d84-d0)

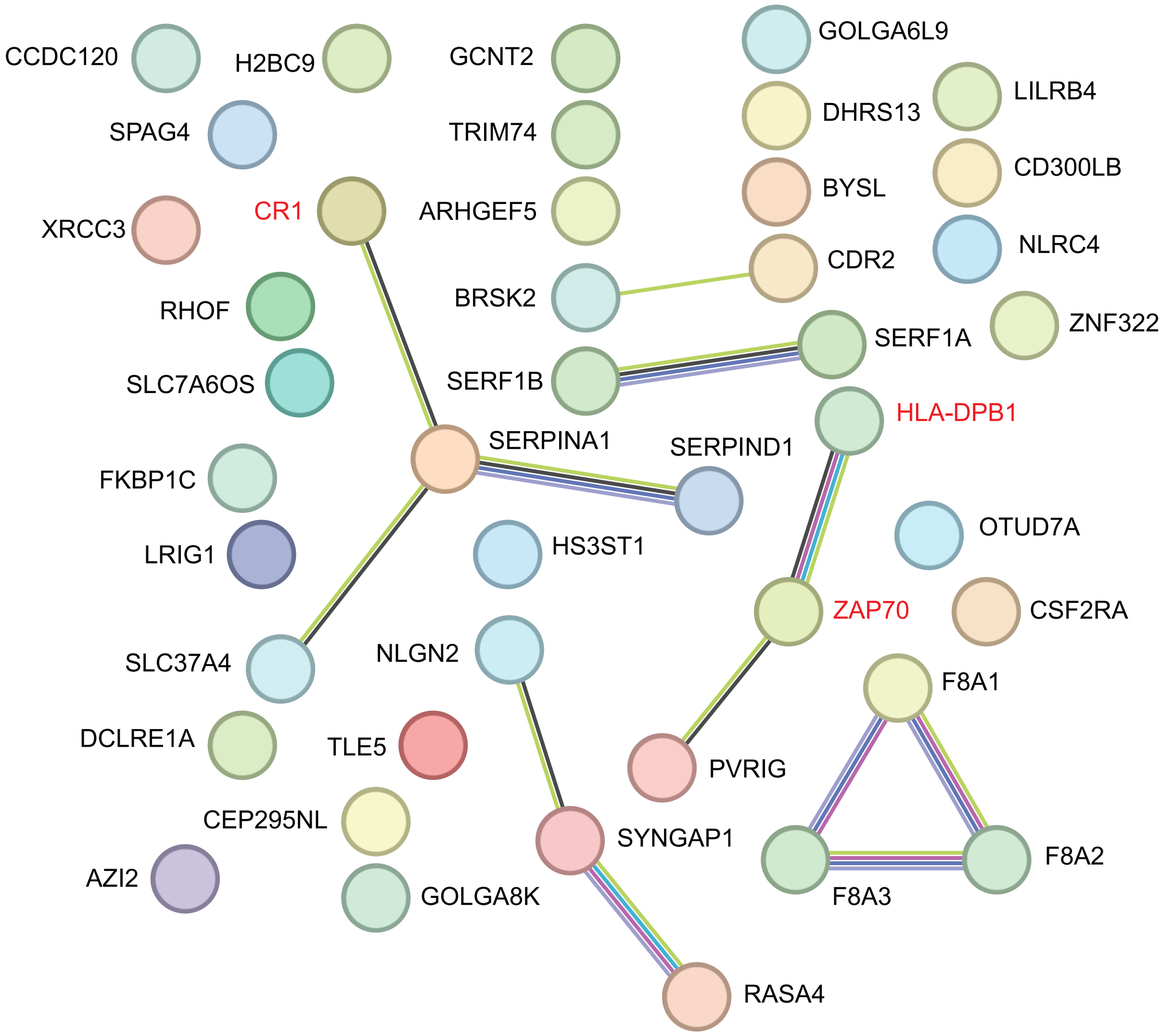
CCDC120
H2BC9
GCNT2
GOLGA6L9
DHRS13
LILRB4
SPAG4
TRIM74
CD300LB
BYSL
XRCC3
CR1
ARHGEF5
NLRC4
CDR2
BRSK2
RHOF
ZNF322
SERF1A
SLC7A6OS
SERF1B
HLA-DPB1
SERPINA1
SERPIND1
FKBP1C
OTUD7A
LRIG1
HS3ST1
ZAP70
CSF2RA
SLC37A4
NLGN2
F8A1
DCLRE1A
TLE5
PVRIG
CEP295NL
SYNGAP1
F8A2
AZI2
F8A3
GOLGA8K
RASA4

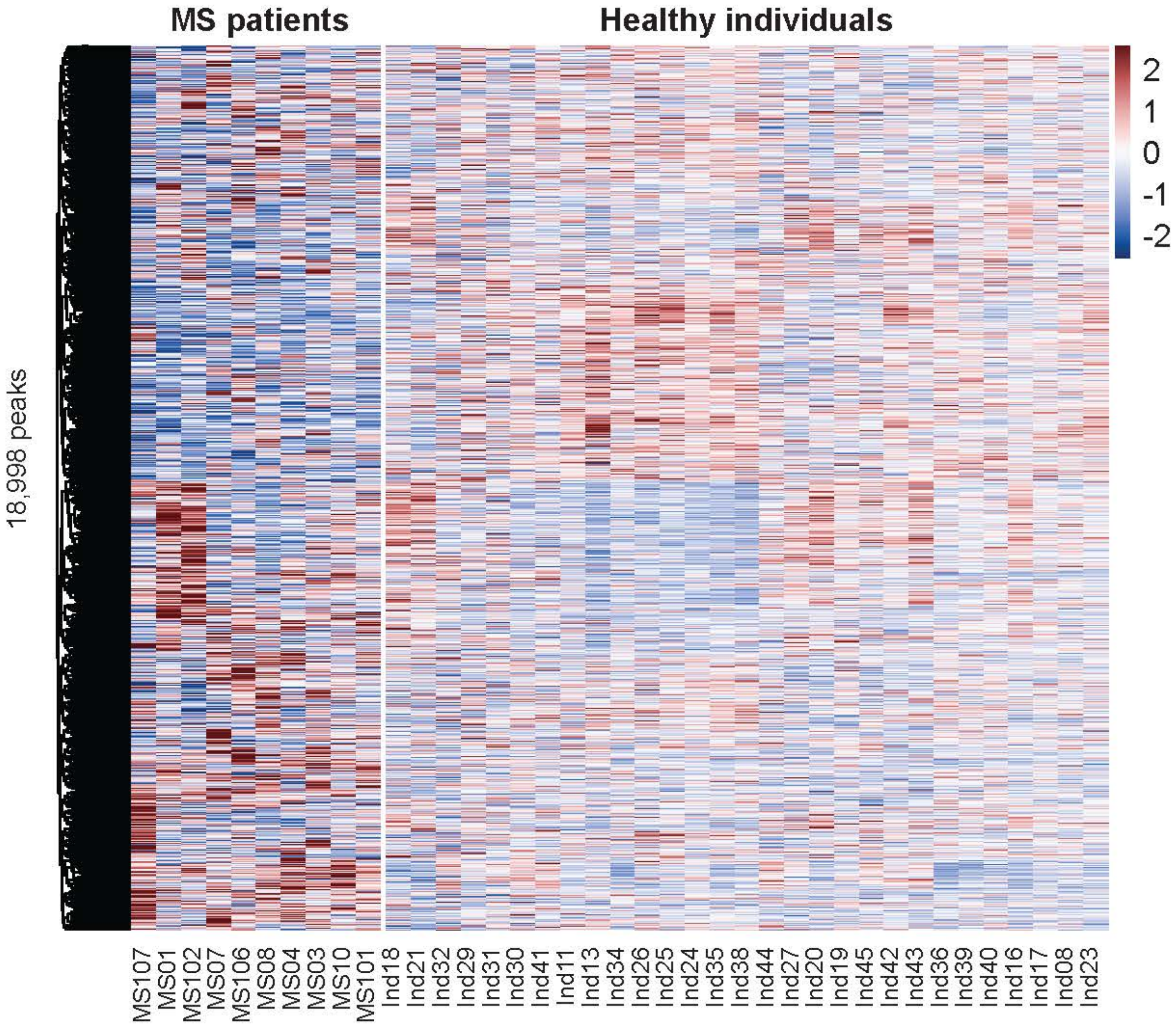
MS patients
Healthy individuals
18,998 peaks
2
1
0
-1
-2
MS107
MS01
MS102
MS07
MS106
MS08
MS04
MS03
MS10
MS101
Ind18
Ind21
Ind32
Ind29
Ind31
Ind30
Ind41
Ind11
Ind13
Ind34
Ind26
Ind25
Ind24
Ind35
Ind38
Ind44
Ind27
Ind20
Ind19
Ind45
Ind42
Ind43
Ind36
Ind39
Ind40
Ind16
Ind17
Ind08
Ind23

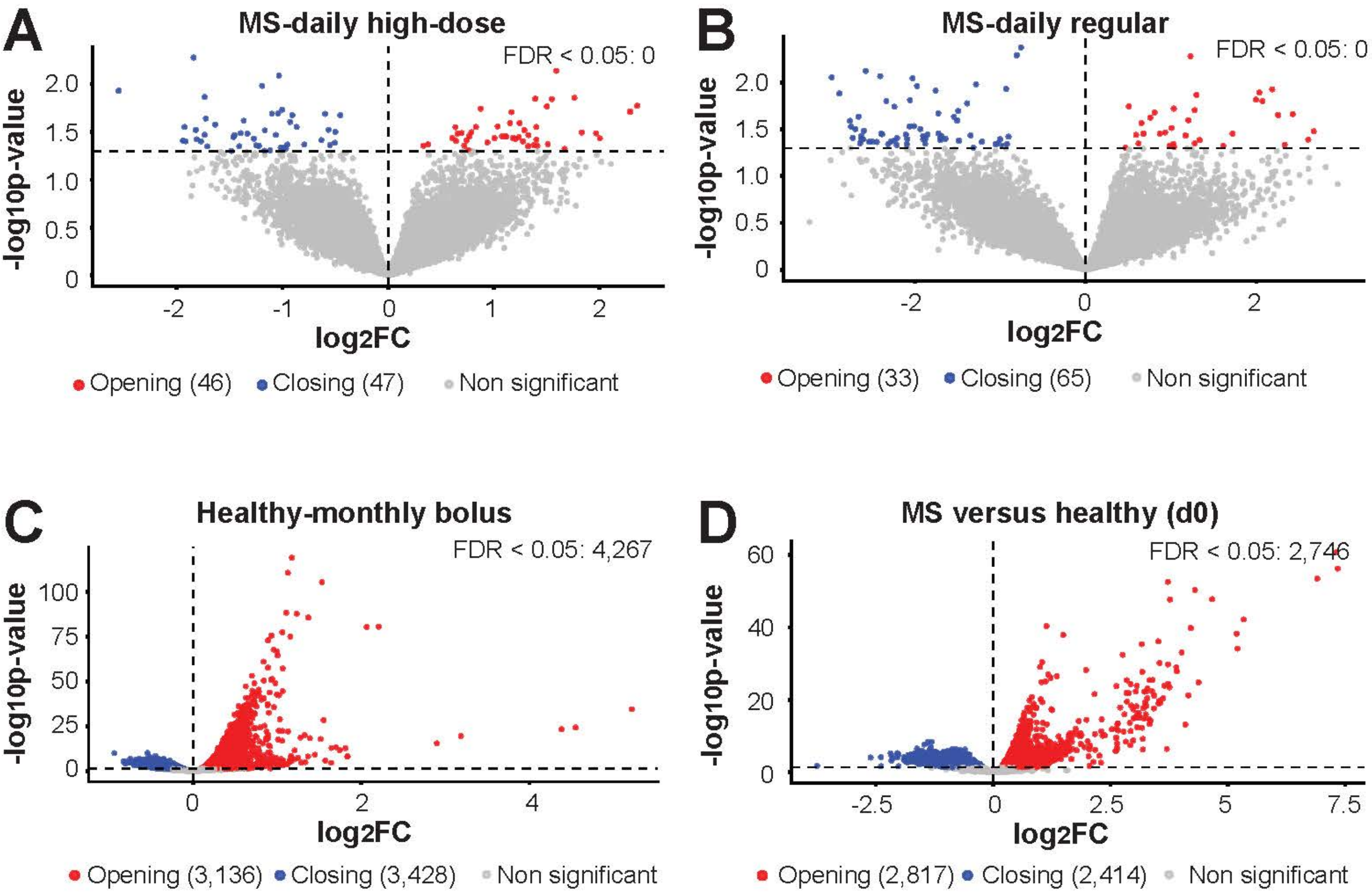
A
MS-daily high-dose
FDR < 0.05: 0
-log10p-value
log2FC
Opening (46) Closing (47) Non significant
B
MS-daily regular
FDR < 0.05: 0
-log10p-value
log2FC
Opening (33) Closing (65) Non significant
C
Healthy-monthly bolus
FDR < 0.05: 4,267
-log10p-value
log2FC
Opening (3,136) Closing (3,428) Non significant
D
MS versus healthy (d0)
FDR < 0.05: 2,746
-log10p-value
log2FC
Opening (2,817) Closing (2,414) Non significant
